# Supplementary material for: Atomic-level insight into super-efficient electrocatalytic oxygen evolution on iron and vanadium co-doped nickel (oxy)hydroxide
Source: Nat Commun. 2018 Jul 23;9:2885. doi: 10.1038/s41467-018-05341-y (PMC6056503; doi:10.1038/s41467-018-05341-y)
Supplement: Supplementary file 1 — Supplementary Information [file 41467_2018_5341_MOESM1_ESM.pdf]

Supplementary Information

Atomic-level insight into super-efficient electrocatalytic oxygen evolution on iron and vanadium co-doped nickel (oxy)hydroxide

Jian Jiang⋆, Fanfei Sun⋆, Si Zhou, Wei Hu, Hao Zhang, Jinchao Dong, Zheng Jiang, Jijun Zhao, Jianfeng Li, Wensheng Yan* & Mei Wang*

⋆These authors contributed equally to this work.

*Corresondence to: symbueno@dlut.edu.cn (M.W.), ywsh2000@ustc.edu.cn (W.Y.)

Supplementary Figures

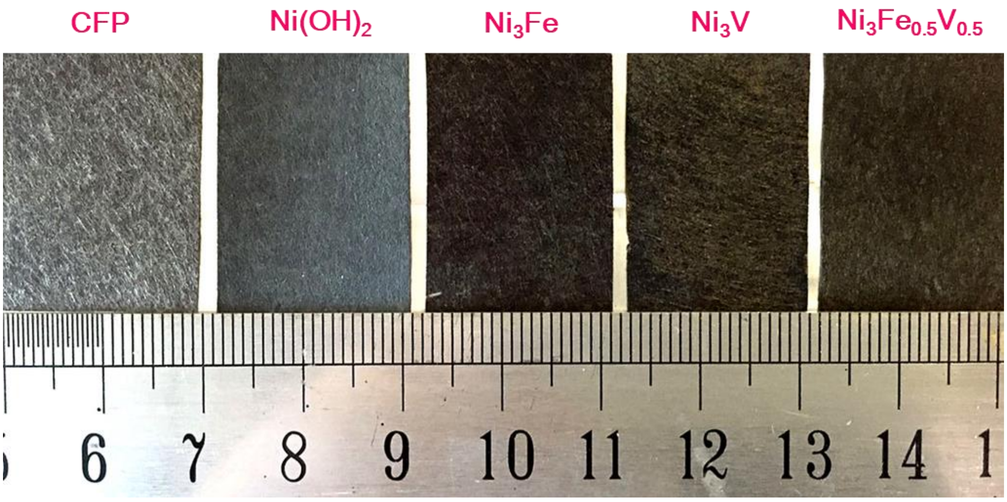

Supplementary Fig. 1 Digital photos of as-prepared $Ni_3Fe_{0.5}V_{0.5}$, Ni_3Fe, Ni_3V, and $Ni(OH)_2$ catalysts on CFP substrates, as well as a pristine CFP.

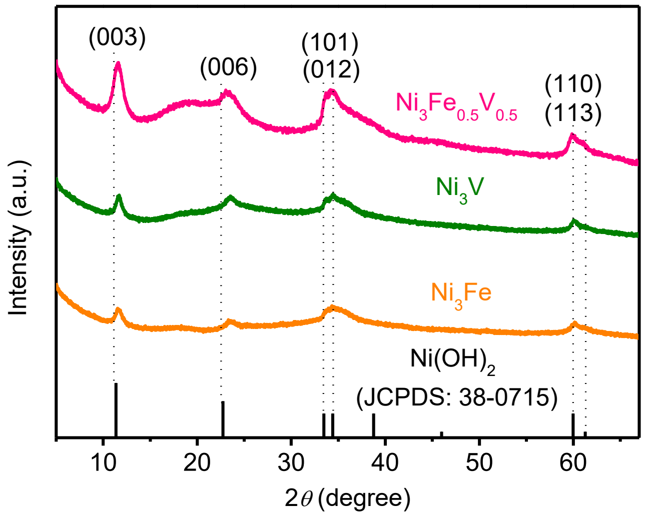

Supplementary Fig. 2 PXRD patterns of $Ni_3Fe_{0.5}V_{0.5}$, Ni_3Fe, and Ni_3V (oxy)hydroxide catalysts compared with the data of α-$Ni(OH)_2$ from JCPDS Card No. 38-0715.

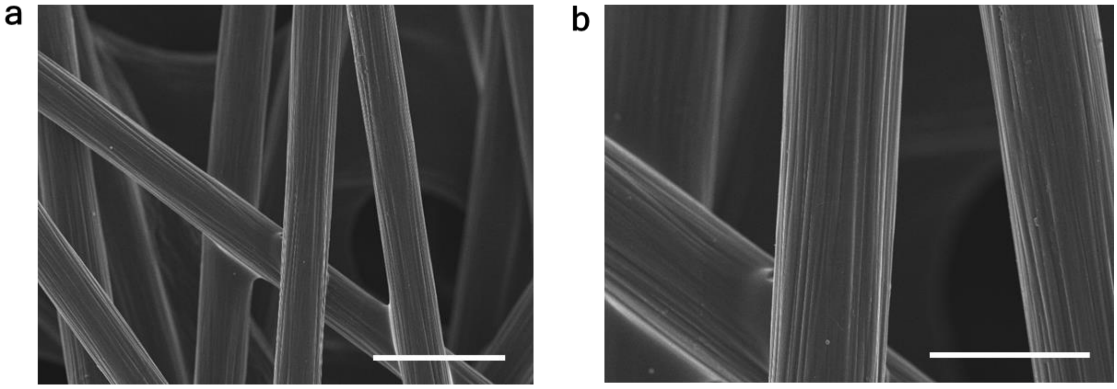

Supplementary Fig. 3 SEM images of a pristine CFP substrate. Scale bar, 20 μm in **a** and 10 μm in **b**.

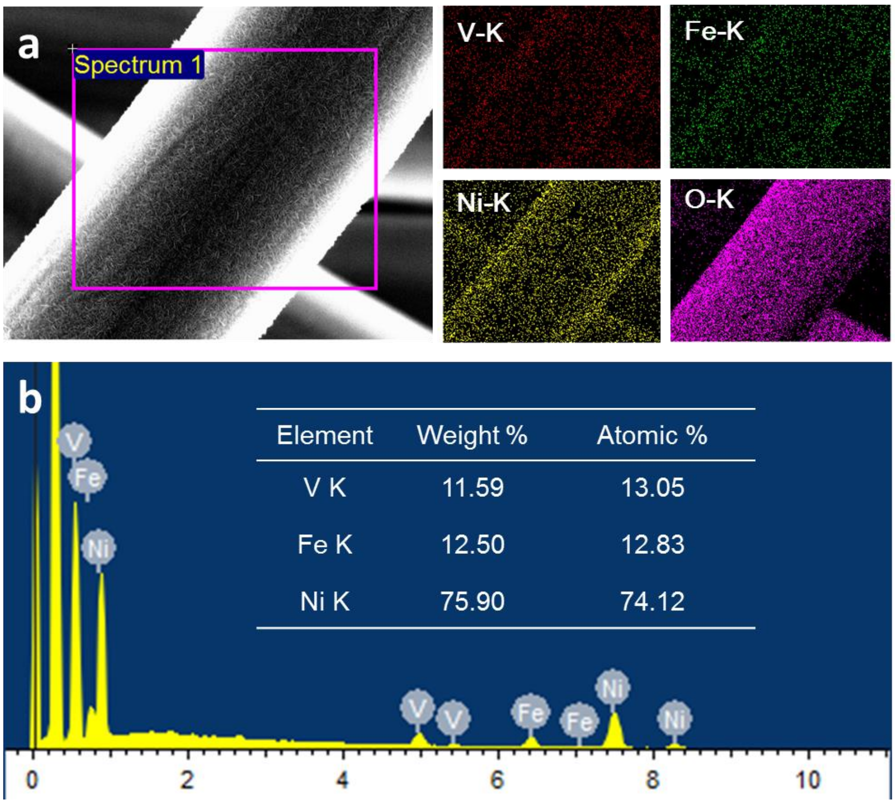

Supplementary Fig. 4 SEM-EDX elemental mapping analysis of the as-prepared $Ni_3Fe_{0.5}V_{0.5}$/CFP electrode. **a** SEM image of $Ni_3Fe_{0.5}V_{0.5}$/CFP and corresponding EDX elemental mappings of V, Fe, Ni, and O in the pink frame of **a**. **b** EDX of the $Ni_3Fe_{0.5}V_{0.5}$ (oxy)hydroxide relative to the pink frame of **a**. Inset of **b** is the corresponding weight and atomic ratio of V, Fe, and Ni elements.

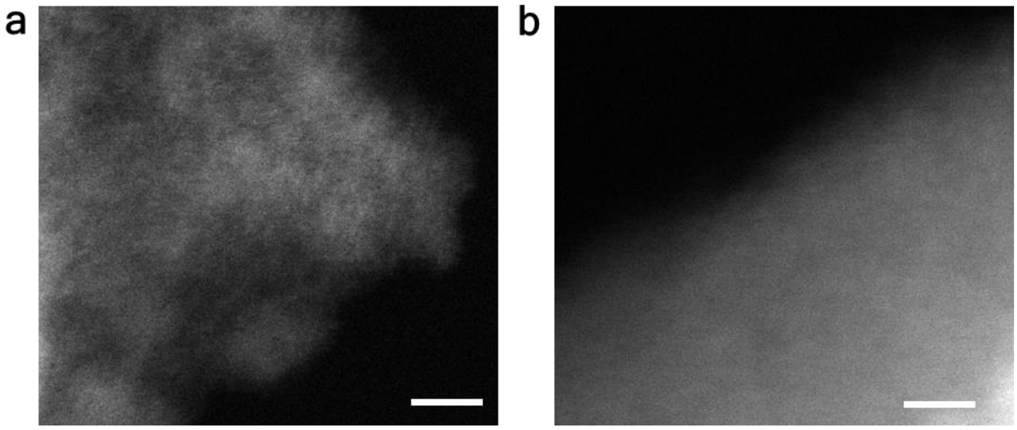

Supplementary Fig. 5 a,b Aberration-corrected HAADF-STEM images of the $Ni_3Fe_{0.5}V_{0.5}$ NSs. No single atoms, clusters or small particles of Fe and V species are observed from the HAADF-STEM images of ultrathin $Ni_3Fe_{0.5}V_{0.5}$ NSs. Scale bar, 2 nm in **a** and 1 nm in **b**.

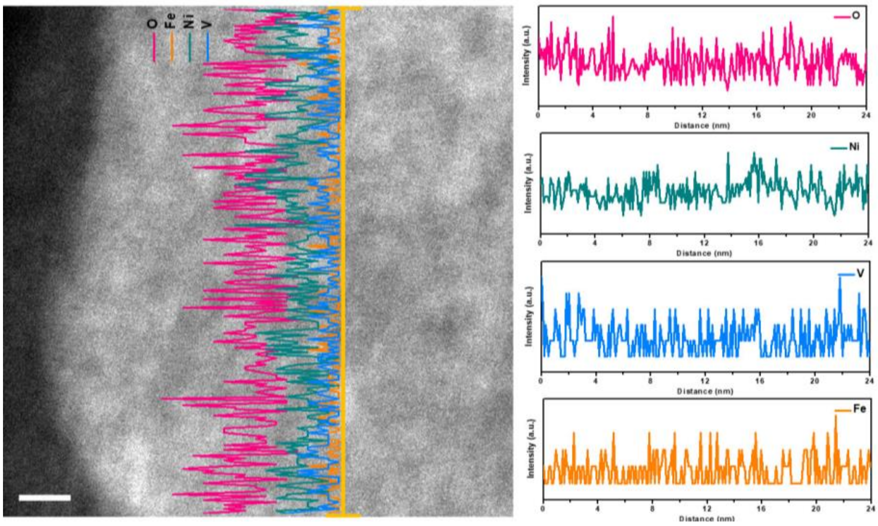

Supplementary Fig. 6 Aberration-corrected HAADF-STEM EDX linear scanning compositional analysis of the $Ni_3Fe_{0.5}V_{0.5}$ NSs. HAADF-STEM image of $Ni_3Fe_{0.5}V_{0.5}$ NSs and corresponding EDX linear scanning compositional analyses of O, Ni, V, and Fe along the yellow line in the left image. Scale bar, 2 nm.

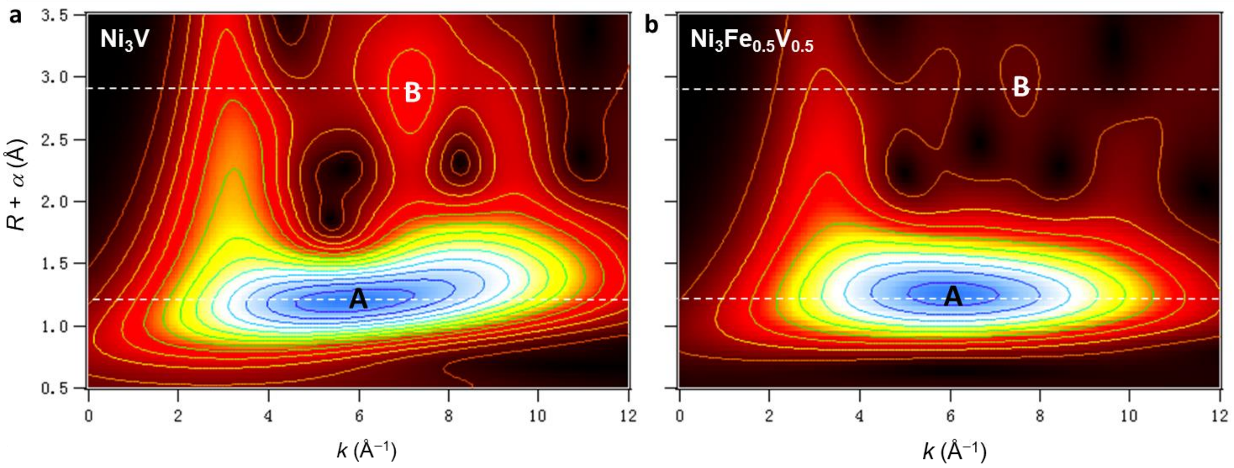

Supplementary Fig. 7 WT-EXAFS of as-prepared Ni_3V (**a**) and $Ni_3Fe_{0.5}V_{0.5}$ (**b**) catalysts.

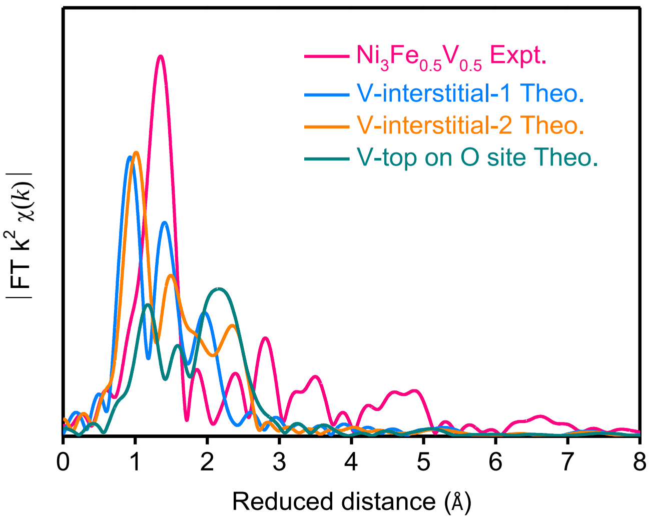

Supplementary Fig. 8 Comparison of the experimental FT curves of V *K*-edge EXAFS $k^2\chi(k)$ functions with the theoretical spectra calculated based on V located at the top or in the interstitial of the NiFe LDH layers.

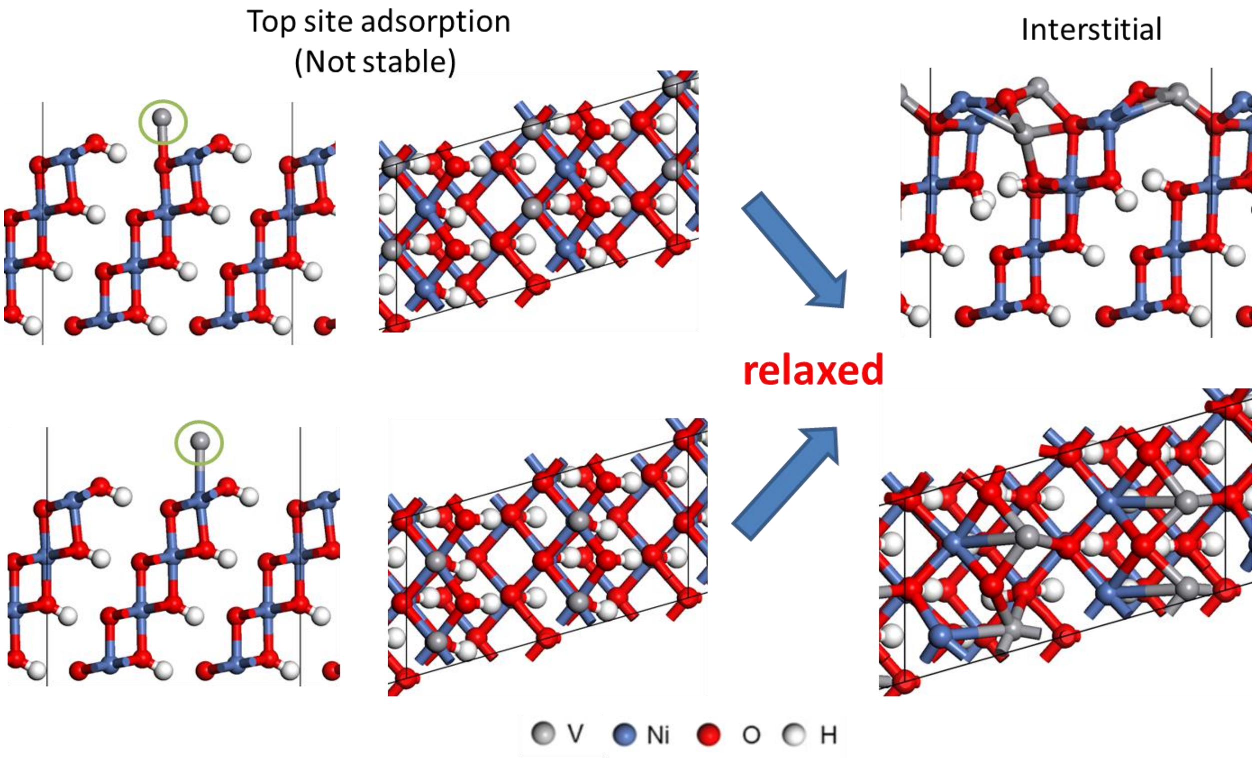

Supplementary Fig. 9 Structural models of different V sites (top site adsorption and interstitial site). The H, O, V, and Ni atoms are shown in white, red, grey, and blue colors, respectively.

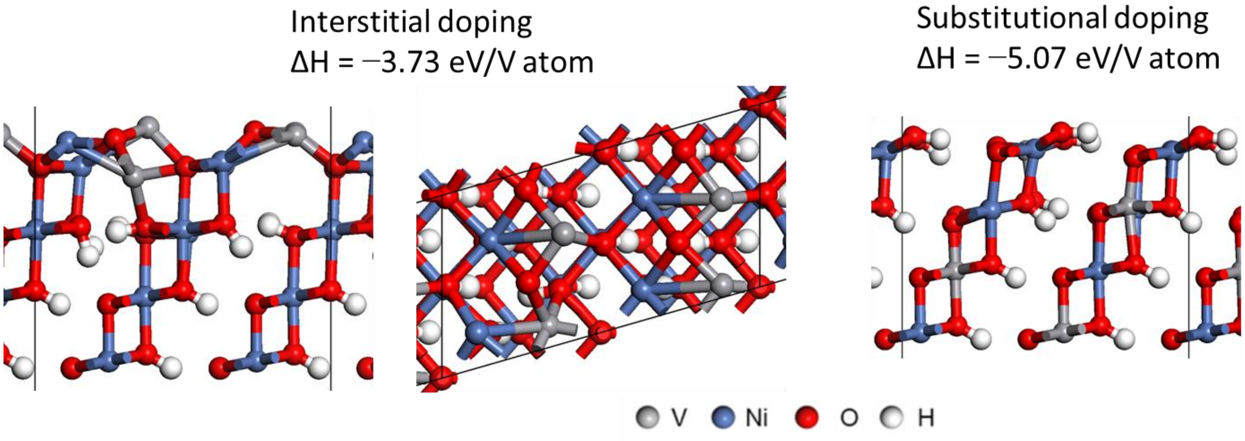

Supplementary Fig. 10 Formation energies of interstitial and substitutional doping structural models. Δ*H* is referred to the energy of the perfect NiOOH as well as bulk Ni and V solids. The H, O, V, and Ni atoms are shown in white, red, grey, and blue colors, respectively.

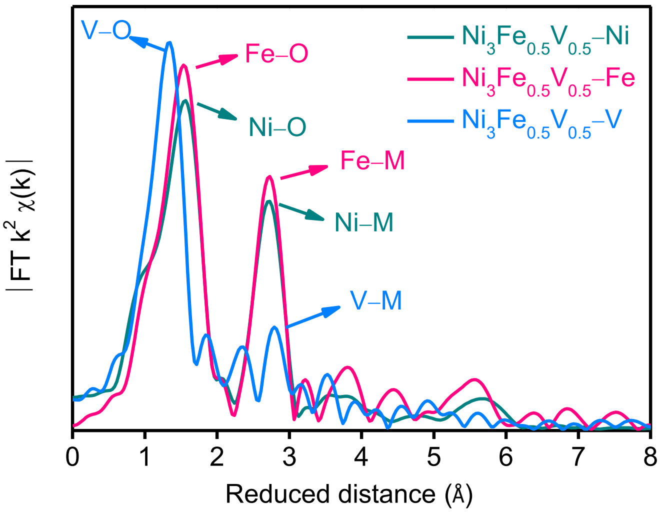

Supplementary Fig. 11 Comparison of FT-EXAFS spectra of the as-prepared $Ni_3Fe_{0.5}V_{0.5}$ at the Ni, Fe and V *K*-edges. The **k**-ranges of the FT are 3.0–12.4 $Å^{-1}$ for Ni and Fe *K*-edges and 3.0–12.5 $Å^{-1}$ for V *K*-edge, and the phase-shifts are not corrected.

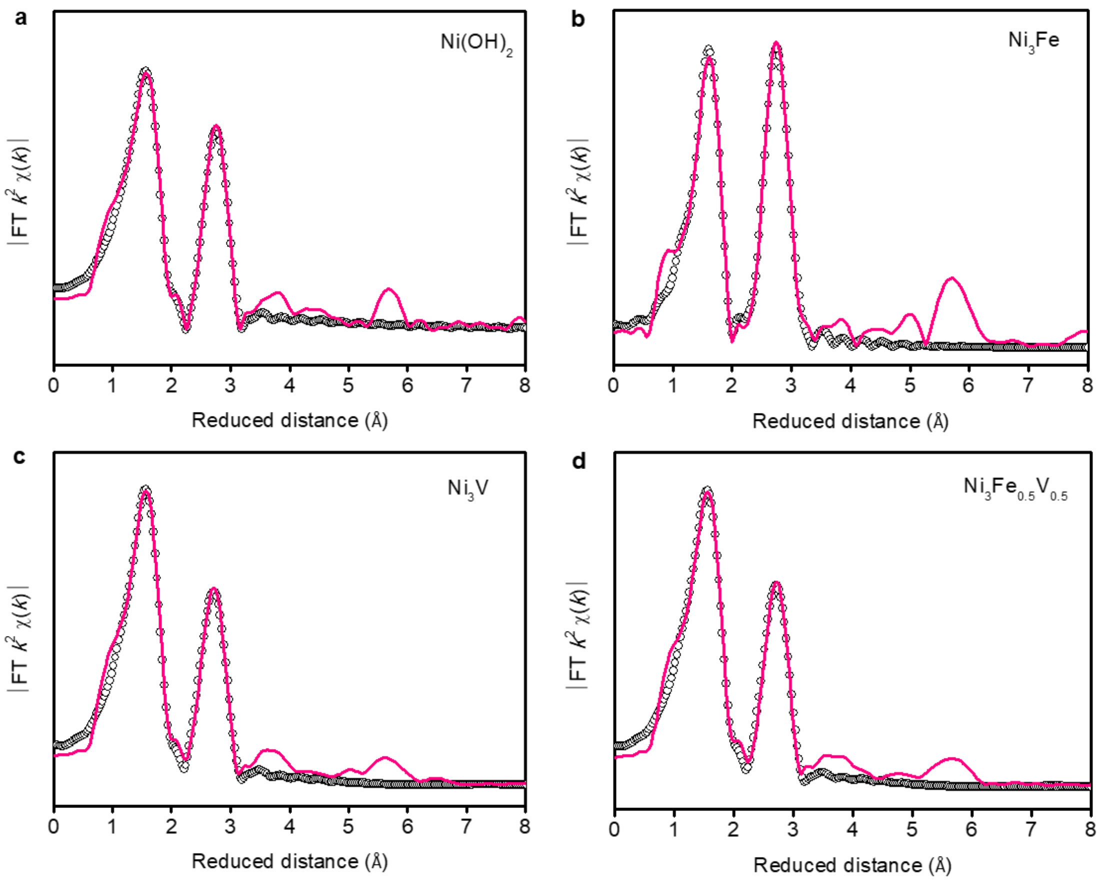

Supplementary Fig. 12 FT-EXAFS spectra and fits of Ni *K*-edge. FT-EXAFS spectra and fits of Ni *K*-edges from (**a**) $Ni(OH)_2$, (**b**) Ni_3Fe, (**c**) Ni_3V, and (**d**) $Ni_3Fe_{0.5}V_{0.5}$ catalysts. The black circles represent the fitting values.

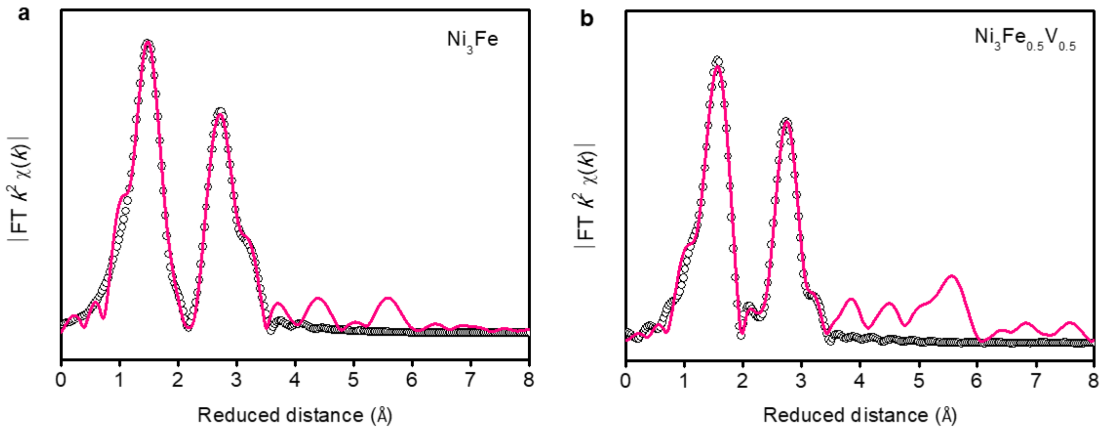

Supplementary Fig. 13 FT-EXAFS spectra and fits of Fe *K*-edge. FT-EXAFS spectra and fits of Fe *K*-edges from (**a**) Ni_3Fe and (**b**) $Ni_3Fe_{0.5}V_{0.5}$ catalysts. The black circles represent the fitting values.

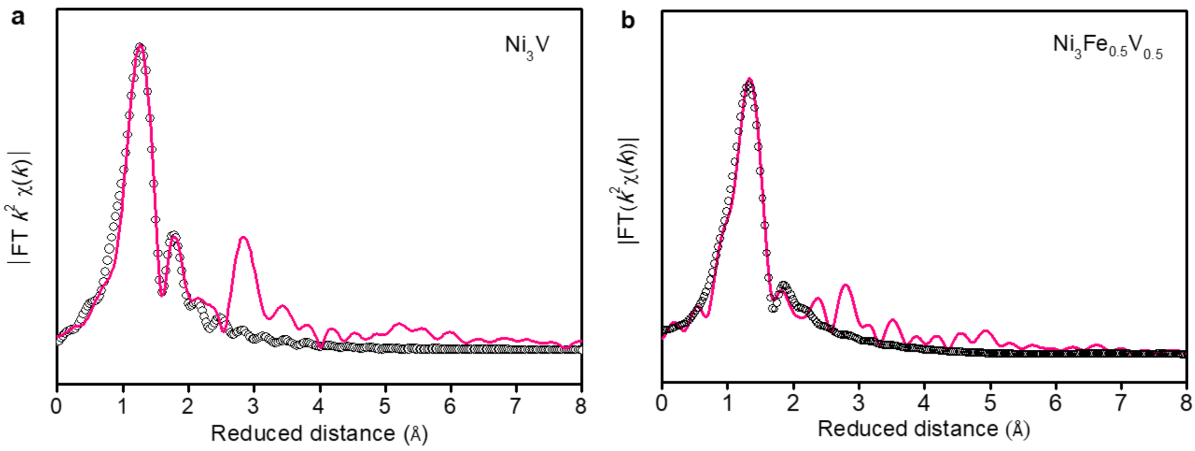

Supplementary Fig. 14 FT-EXAFS spectra and fits of V *K*-edge. FT-EXAFS spectra and fits of V *K*-edges from (**a**) Ni_3Fe and (**b**) $Ni_3Fe_{0.5}V_{0.5}$ catalysts. The black circles represent the fitting values.

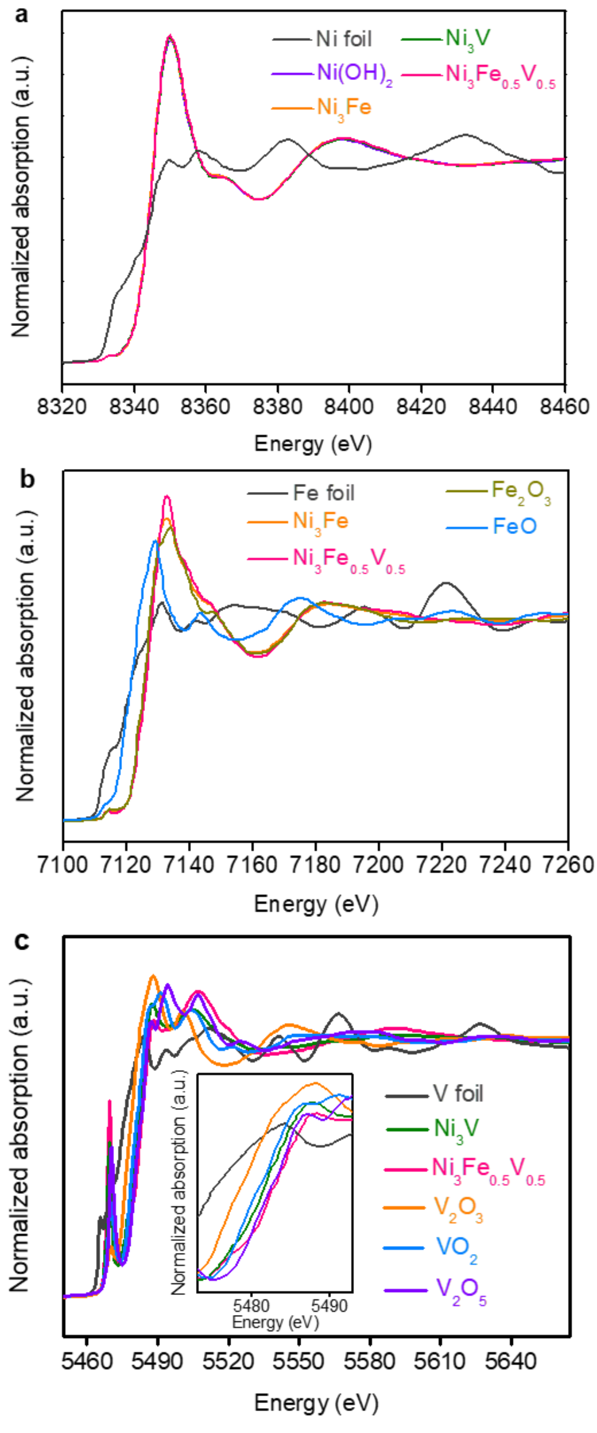

Supplementary Fig. 15| XANES spectra at (**a**) Ni *K*-edge, (**b**) Fe *K*-edge, and (**c**) V *K*-edge from as-prepared catalysts and Fe_2O_3, FeO, V_2O_3, VO_2, and V_2O_5 references.

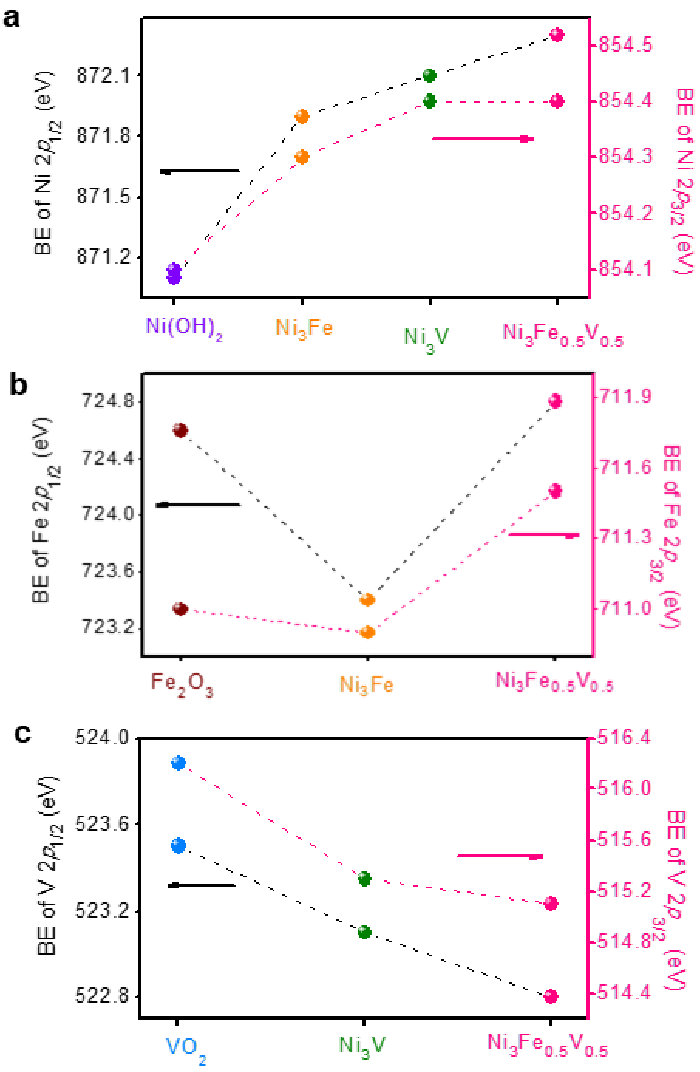

Supplementary Fig. 16 Plots showing changes in binding energy. Changes in binding energy of (**a**) Ni 2*p*, (**b**) Fe 2*p*, and (**c**) V 2*p* for the as-prepared $Ni_3Fe_{0.5}V_{0.5}$, Ni_3Fe, Ni_3V, and pure $Ni(OH)_2$ catalysts, together with Fe_2O_3 and VO_2 as unary metal oxide references. The dashed lines in **a**,**b**, and **c** are guide for eyes.

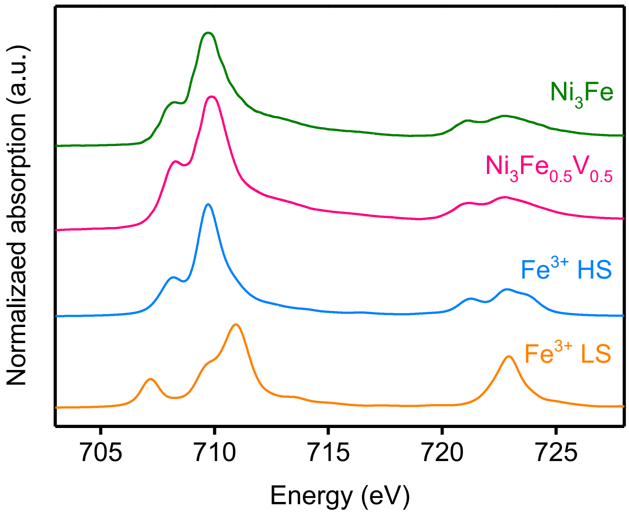

Supplementary Fig. 17 XANES spectra at V *L*-edges from the as-prepared Ni_3V and $Ni_3Fe_{0.5}V_{0.5}$ catalysts and the calculated spectra of high spin (HS) and low spin (LS) Fe^{3+}.

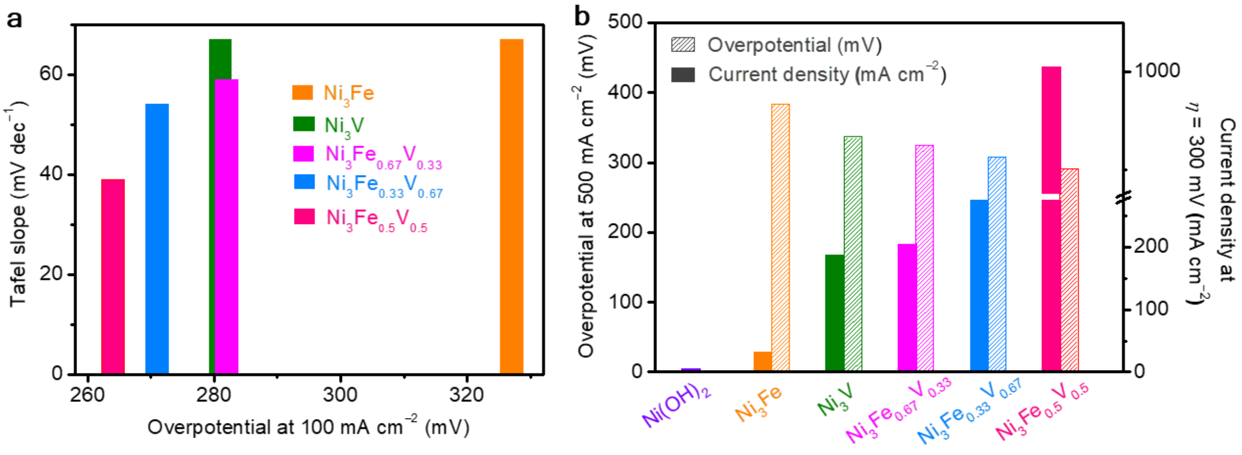

Supplementary Fig. 18 Comparison of OER activities of the as-fabricated Ni-based (oxy)hydroxide catalysts. **a** Comparison of overpotentials (*η*) at 100 mA cm^{-2} current density and Tafel slopes for Ni_3Fe, Ni_3V, $Ni_3Fe_{0.67}V_{0.33}$, $Ni_3Fe_{0.5}V_{0.5}$, and $Ni_3Fe_{0.33}V_{0.67}$ (oxy)hydroxides. **b** Comparison of overpotentials (*η*) at 500 mA cm^{-2} current density and current density (*J*) at 300 mV overpotential for $Ni(OH)_2$, Ni_3Fe, Ni_3V, $Ni_3Fe_{0.67}V_{0.33}$, $Ni_3Fe_{0.33}V_{0.67}$, and $Ni_3Fe_{0.5}V_{0.5}$ (oxy)hydroxides. The solid and grid frames are corresponding to the right and left *y*-axis, respectively.

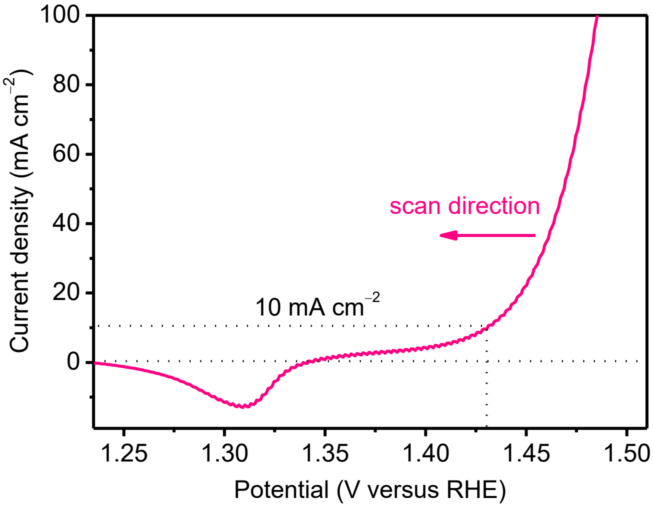

Supplementary Fig. 19 LSV of $Ni_3Fe_{0.5}V_{0.5}$/CFP for OER. LSV curve of $Ni_3Fe_{0.5}V_{0.5}$/CFP in O_2-saturated 1 M KOH at a scan rate of 5 mV s^{-1}. The LSV was scanned from positive to negative direction to exclude the influence of the Ni^{2+}/Ni^{3+} oxidation event on the catalytic current, and thus to identify the overpotential at low current density.

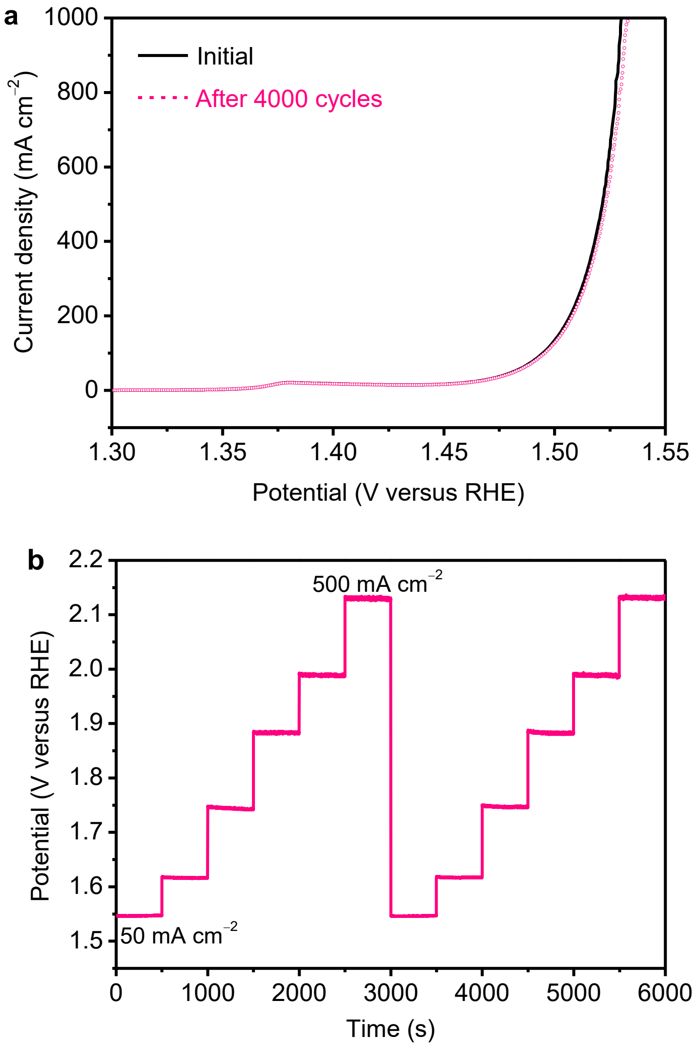

Supplementary Fig. 20 Polarization curves and multi-current step test of $Ni_3Fe_{0.5}V_{0.5}$. **a** Polarization curves before and after CV testing of 4000 cycles at a scan rate of 100 mV s^{-1} in 1 M KOH. **b** Two-cycle multi-current step test of $Ni_3Fe_{0.5}V_{0.5}$/CFP at current densities of 50, 100, 200, 300, 400 and 500 mA cm^{-2} without *iR*-correction.

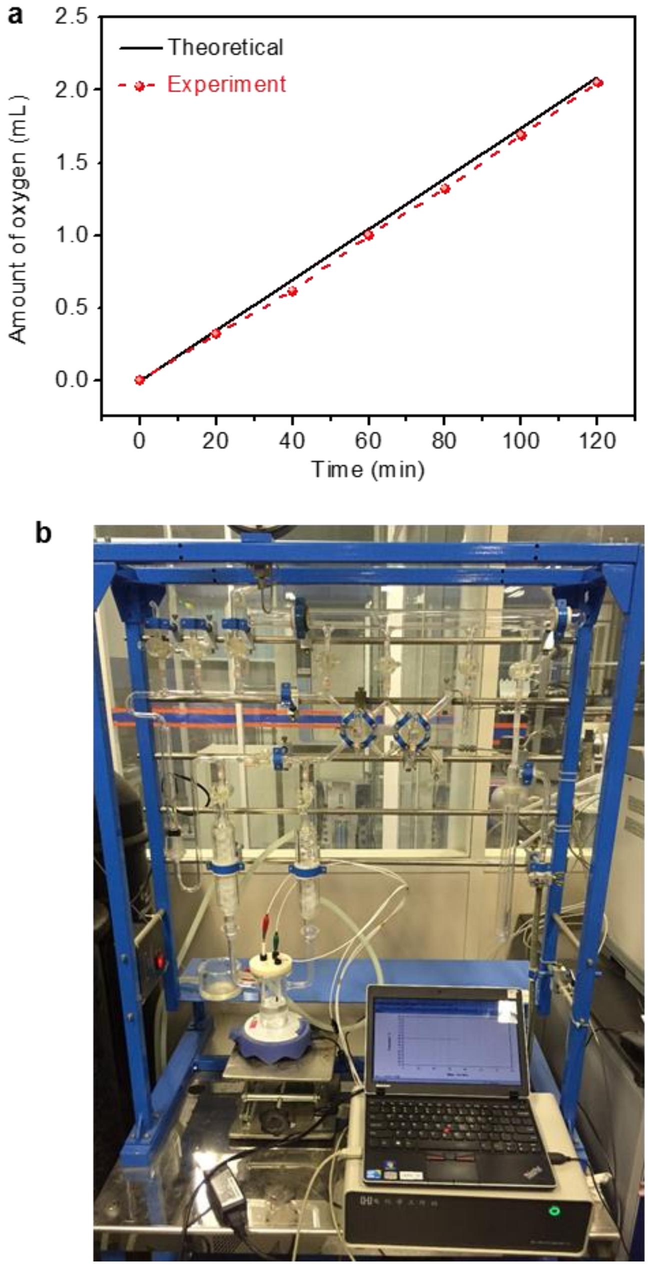

Supplementary Fig. 21 Measurement of Faradaic efficiency. **a** Faradaic efficiency of $Ni_3Fe_{0.5}V_{0.5}$/CFP was evaluated from chronopotentiometric experiments operated in 1 M KOH at a constant current density of 10 mA cm^{-2} for 2 h. **b** Illustration of on-line analysis system that combines electrochemical equipment and gas chromatography together for measurement of Faradaic efficiency. The sealed electrochemical cell with a three-electrode configuration was linked to a closed circulation system for gas analysis. The amount of oxygen experimentally generated was compared to the theoretically calculated oxygen based on the charge consumed, giving a Faradaic efficiency close to 100%.

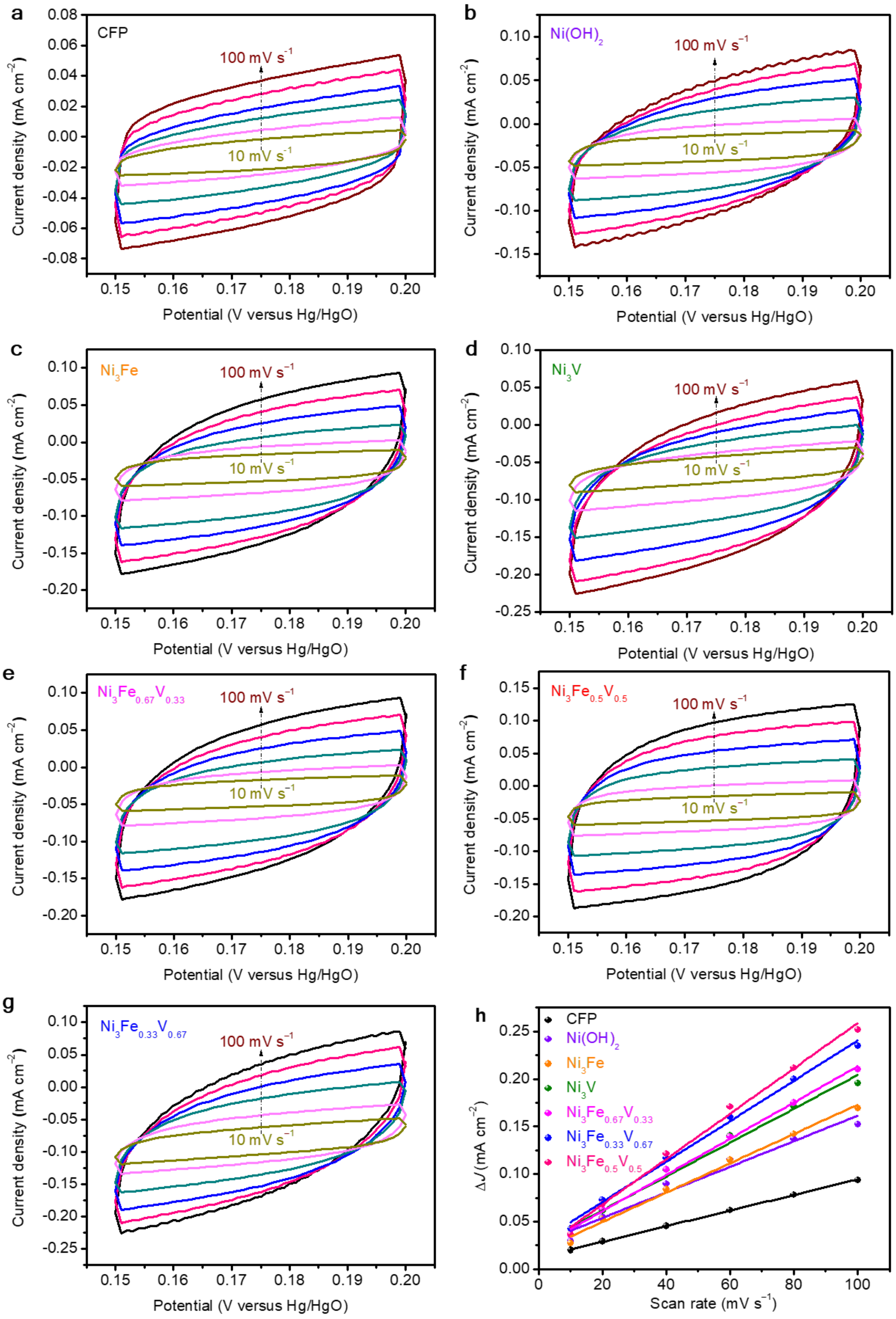

Supplementary Fig. 22 Cyclic voltammograms of the as-fabricated Ni-based (oxy)hydroxide catalysts. Cyclic voltammograms of (**a**) CFP, (**b**) $Ni(OH)_2$, (**c**) Ni_3Fe, (**d**) Ni_3V, (**e**) $Ni_3Fe_{0.67}V_{0.33}$, (**f**) $Ni_3Fe_{0.5}V_{0.5}$, and (**g**) $Ni_3Fe_{0.33}V_{0.67}$ (oxy)hydroxide catalysts in 1 M KOH at different scan rates (10, 20, 40, 60, 80, 100 mV s^{-1}) in the non-Faradaic potential region (0.15–0.2 V versus Hg/HgO). **h** Capacitive current density ($\Delta J = J_a - J_c$) at 0.175 V versus Hg/HgO as a function of scan rate.

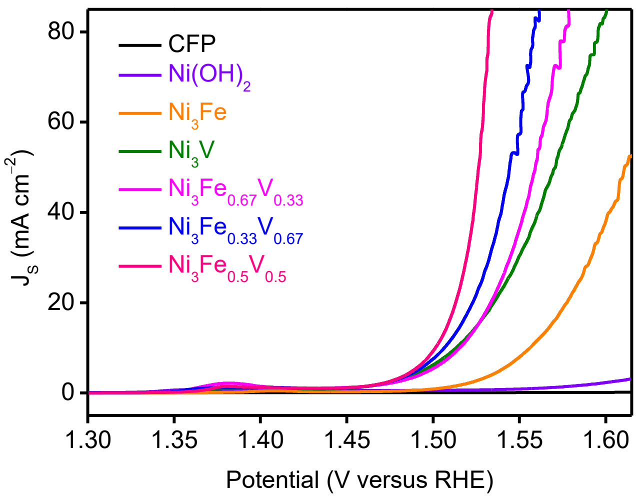

Supplementary Fig. 23 Specific OER activity (J_S) of as-prepared Ni-based (oxy)hydroxide catalysts. LSV curves are normalized by the RFs, which indicates the higher specific activity of the $Ni_3Fe_{0.5}V_{0.5}$ (oxy)hydroxide than the OER activities of $Ni(OH)_2$, Ni_3Fe and Ni_3V (oxy)hydroxides.

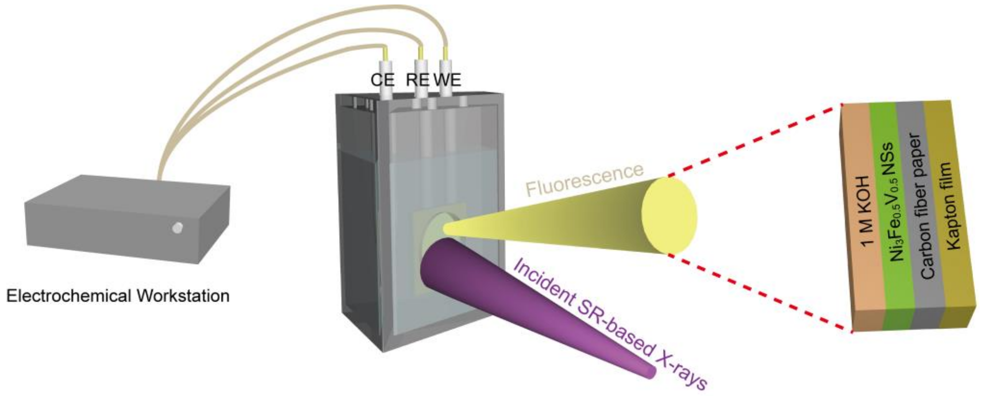

***In-situ* XAS equipment**

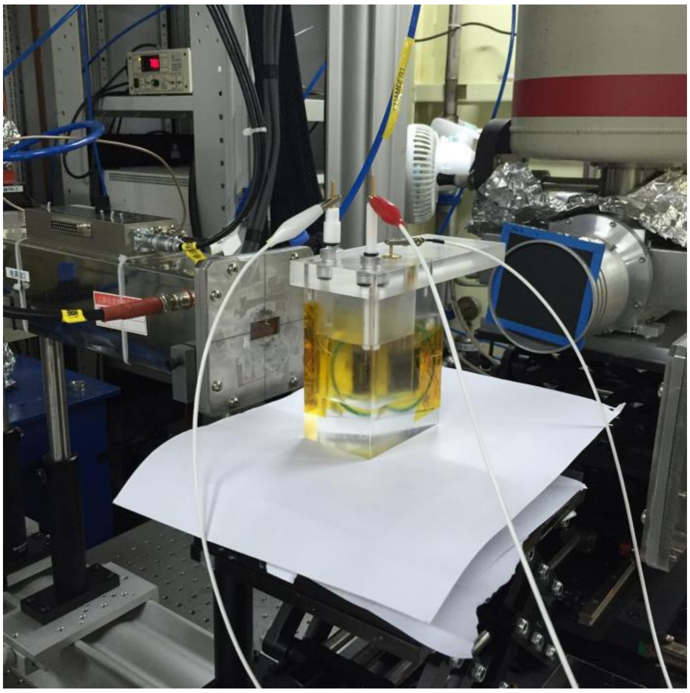

Supplementary Fig. 24 Equipment used for *in-situ* XAS measurements. Schematic for the equipment used for *in-situ* XAS measurements (top). Digital photo taken during *in-situ* XAS measurement (bottom).

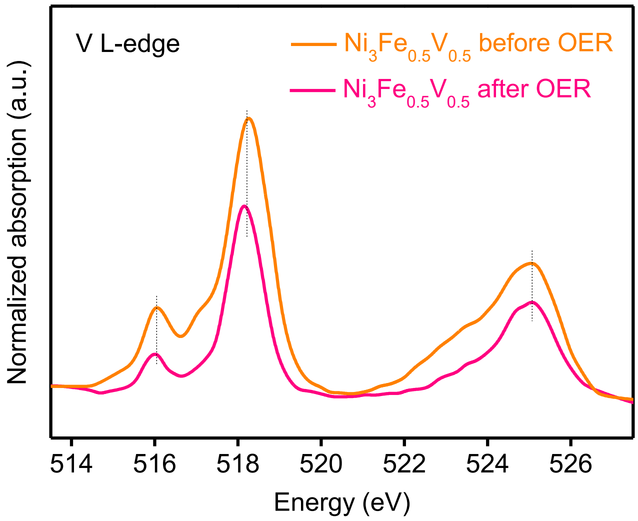

Supplementary Fig. 25 XANES spectra at V *L*-edge. Soft XANES spectra at V *L*-edge for $Ni_3Fe_{0.5}V_{0.5}$ before and after OER at 1.75 V in 1 M KOH.

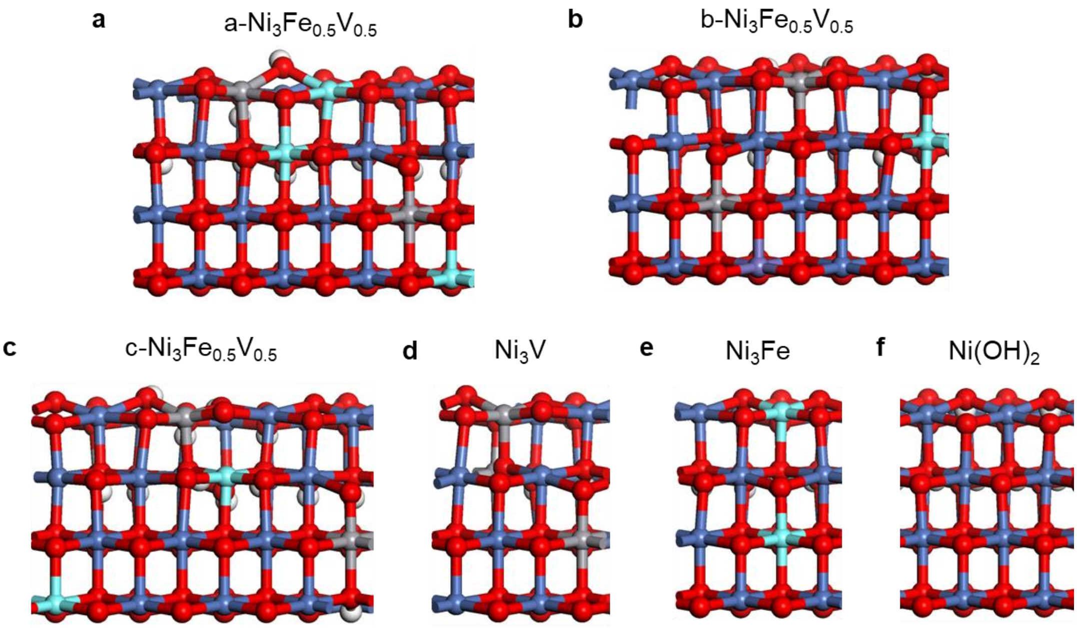

Supplementary Fig. 26 Side views of the (101) surface of Fe and V doped Ni (oxy)hydroxide models for DFT calculations. **a**,**b**,**c** Fe/V co-doped Ni (oxy)hydroxide with Fe and V atoms in different relative positions. **d** V-doped, (**e**) Fe-doped, and (**f**) pure Ni (oxy)hydroxide. The H, O, V, Fe and Ni atoms are shown in white, red, grey, cyan and blue colors, respectively.

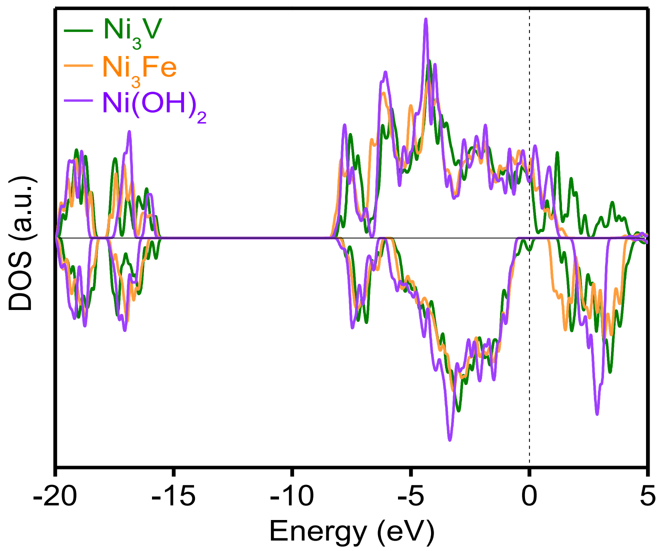

Supplementary Fig. 27 Density of states (DOSs) of V-doped, Fe-doped, and pure Ni (oxy)hydroxides. The Fermi level is shifted to zero.

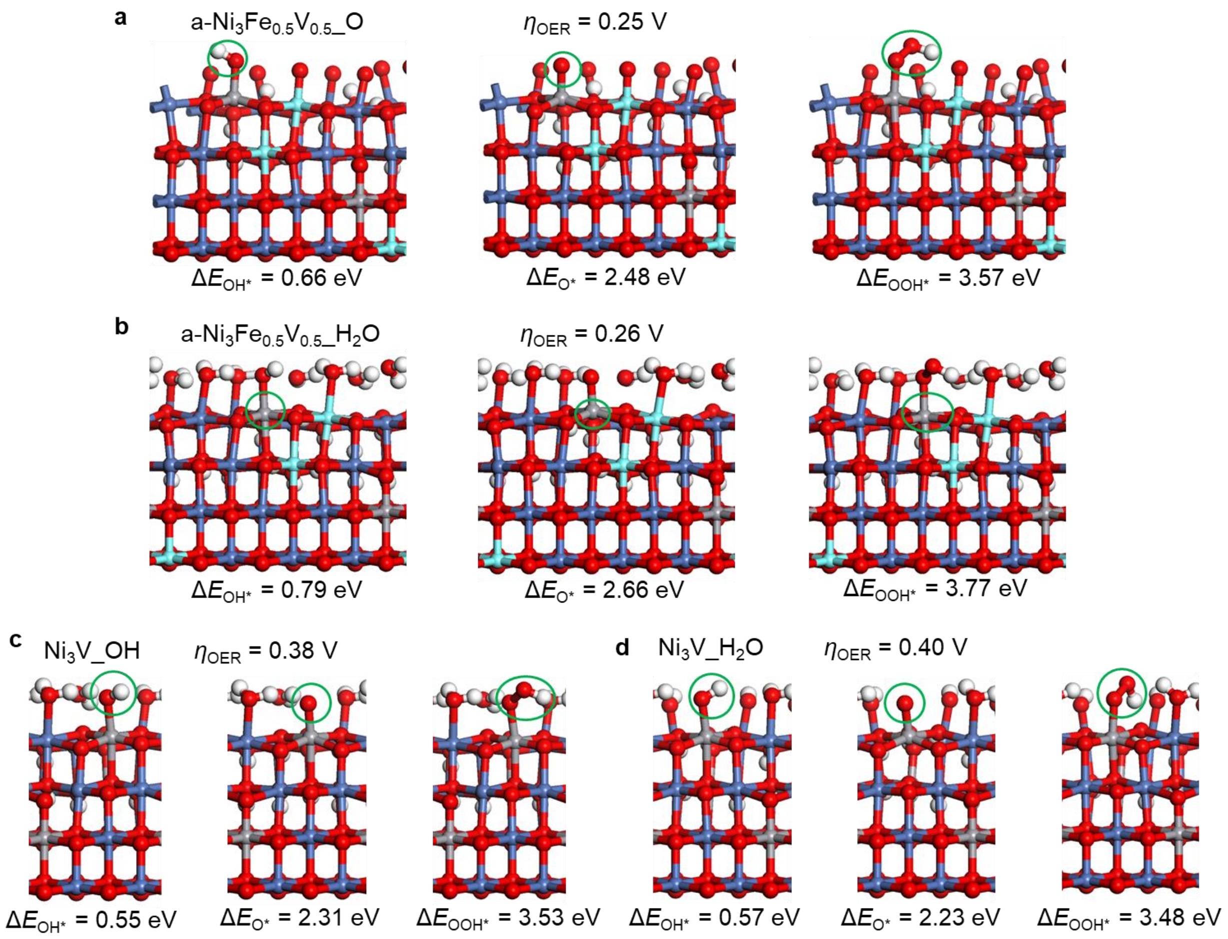

Supplementary Fig. 28 Structures and binding energies of oxygen intermediates. Oxygen intermediates on the V sites of the (101) surface of model a-$Ni_3Fe_{0.5}V_{0.5}$ (oxy)hydroxide covered by (**a**) O and (**b**) H_2O species, and model Ni_3V (oxy)hydroxide covered by (**c**) OH and (**d**) H_2O species. The H, O, V, Fe and Ni atoms are shown in white, red, grey, cyan and blue colors, respectively. The calculated OER overpotentials are indicated for each system.

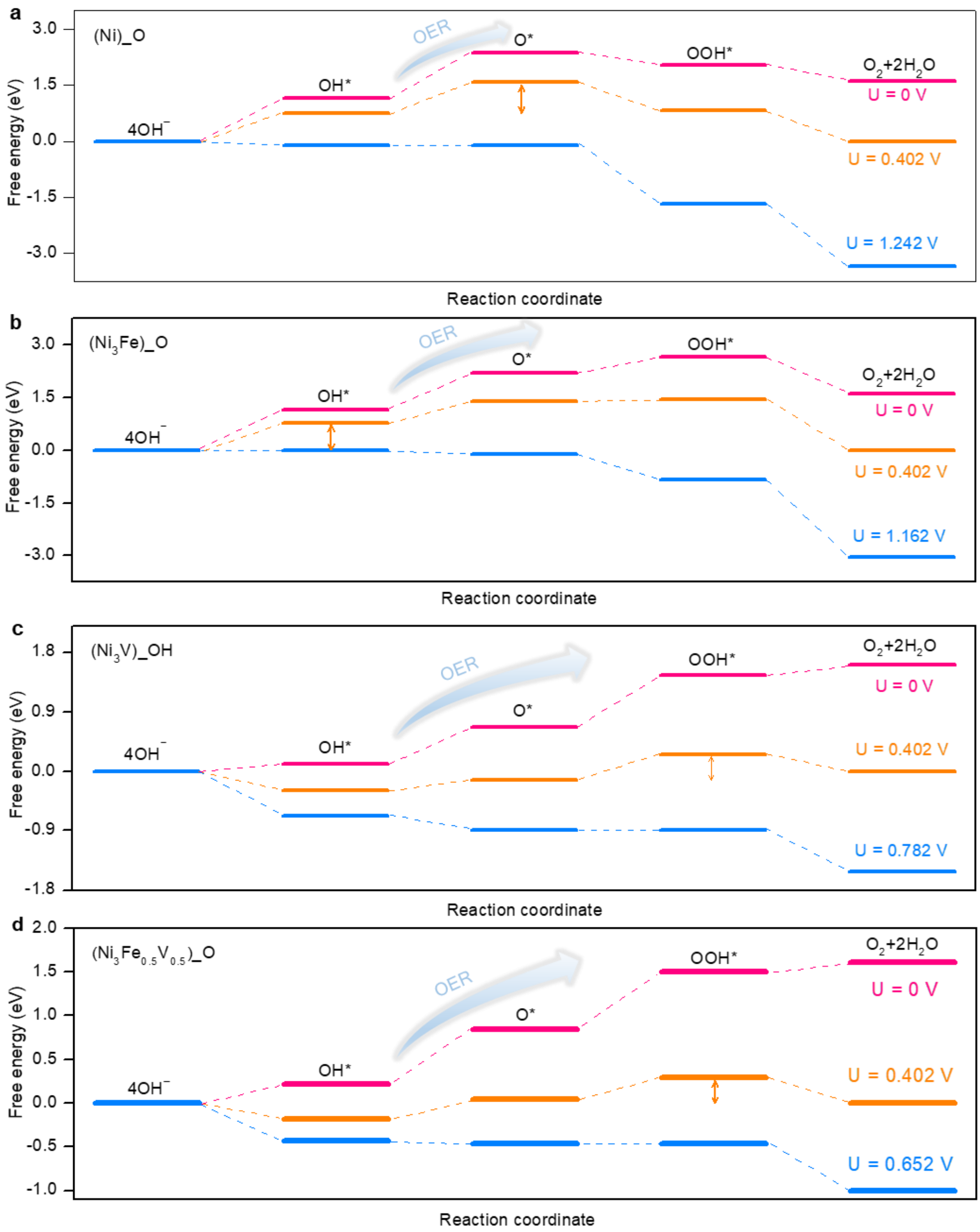

Supplementary Fig. 29 Calculated free-energy diagrams. Calculated free-energy diagrams of OER on the most active sites of (**a**) pure $Ni(OH)_2$, (**b**) Fe-doped, (**c**) V-doped, and (**d**) Fe/V co-doped Ni (oxy)hydroxides in alkaline media (pH = 14 and T = 298 K) at different potentials. The two-way arrows indicate the rate-limiting steps and overpotentials.

Supplementary Tables

Supplementary Table 1. ICP-OES analysis data for the loading amounts and atomic ratios of metals of the as-prepared $Ni_3Fe_{1-x}V_x$ NSs/CFP matrix

$Ni_3Fe_{1-x}V_x$ (oxy)hydroxides ($0 \leq x \leq 1$)	Metal chlorides (mmol) in the precursor solution			Loading amount of metals (mg cm^{-2})			Atomic ratio of metals
	$NiCl_2{\cdot}6H_2O$	$FeCl_3{\cdot}6H_2O$	VCl_3	Ni	Fe	V	Ni:Fe or Ni:V or Ni:Fe:V
$Ni(OH)_2$	0.8	–	–	0.312	–	–	–
Ni_3Fe	0.6	0.2	–	0.205	0.069	–	2.82:1
$Ni_3Fe_{0.67}V_{0.33}$	0.6	0.133	0.067	0.203	0.041	0.0192	9.13:1.92:1
$Ni_3Fe_{0.5}V_{0.5}$	0.6	0.1	0.1	0.204	0.0284	0.0283	6.25:0.91:1
$Ni_3Fe_{0.33}V_{0.67}$	0.6	0.067	0.133	0.204	0.0239	0.0389	4.56:0.56:1
Ni_3V	0.6	–	0.2	0.209	–	0.0644	2.77:1

Supplementary Table 2. Summary the fitting parameters of *ex-situ* Ni *K*-edge EXFAS curves for the as-prepared $Ni_3Fe_{1-x}V_x$ catalysts

Catalyst	Shell	Coordination number (CN)	Bond distance (R (Å))	ΔE_0 (eV)	σ^2 (10^{-3} Å^2)	*R*-factor
$Ni(OH)_2$	Ni−O	6.2 ± 0.4	2.03 ± 0.01	−6.2 ± 0.7	8.0 ± 0.8	0.003
	Ni−Ni	6.0 ± 0.7	3.12 ± 0.01	−0.9 ± 1.1	10.5 ± 0.9	
Ni_3Fe	Ni−O	6.6 ± 0.5	2.04 ± 0.01	−5.6 ± 0.8	8.0 ± 0.9	0.003
	Ni−M	7.4 ± 0.1	3.10 ± 0.01	10.0 ± 1.0	10.0 ± 1.0	
Ni_3V	Ni−O	6.5 ± 0.3	2.03 ± 0.01	−6.0 ± 0.6	8.6 ± 0.7	0.002
	Ni−M	5.4 ± 0.6	3.10 ± 0.01	−6.4 ± 2.0	10.8 ± 0.1	
$Ni_3Fe_{0.5}V_{0.5}$	Ni−O	6.3 ± 0.4	2.03 ± 0.01	−6.1 ± 0.7	8.8 ± 0.8	0.003
	Ni−M	5.1 ± 0.6	3.10 ± 0.01	−0.4 ± 1.1	10.5 ± 1.1	

Note: ΔE_0, inner potential correction; σ^2, Debye–Waller factor to account for both thermal and structural disorders; *R*-factor, indicating the goodness of the fit.

Supplementary Table 3. Summary the fitting parameters of *ex-situ* Fe *K*-edge EXFAS curves for the as-prepared Ni_3Fe and $Ni_3Fe_{0.5}V_{0.5}$ catalysts

Catalyst	Shell	Coordination number (CN)	Bond distance (R (Å))	ΔE_0 (eV)	σ^2 (10^{-3} Å^2)	R-factor
Ni_3Fe	Fe−O	6.4 ± 0.5	2.02 ± 0.01	−1.7 ± 1.2	11.2 ± 1.3	0.002
	Fe−M	5.8 ± 1.2	3.09 ± 0.02	5.4 ± 2.9	12.0 ± 1.7	
	Fe−O1	5.4 ± 2.9	3.72 ± 0.02	−1.7 ± 1.2	6.8 ± 5.4	
$Ni_3Fe_{0.5}V_{0.5}$	Fe−O	6.2 ± 0.3	2.00 ± 0.01	−2.3 ± 0.6	6.2 ± 0.7	0.002
	Fe−M	5.8 ± 0.9	3.10 ± 0.01	0.8 ± 1.4	8.6 ± 1.2	
	Fe−O1	4.0 ± 2.0	3.70 ± 0.02	−2.3 ± 0.6	5.7 ± 5.7	

Supplementary Table 4. Summary the fitting parameters of *ex-situ* V *K*-edge EXFAS curves for the as-prepared Ni_3V and $Ni_3Fe_{0.5}V_{0.5}$ catalysts

Catalyst	Shell	Coordination number (CN)	Bond distance (*R* (Å))	ΔE_0 (eV)	σ^2 (10^{-3} Å^2)	*R*-factor
Ni_3V	V−O1	3.5	1.69 ± 0.01	6.1 ± 1.7	6.6 ± 0.5	0.009
	V−O2	2.5	1.99 ± 0.01	5.0	5.8 ± 1.3	
$Ni_3Fe_{0.5}V_{0.5}$	V−O1	4.6 ± 0.4	1.72 ± 0.01	6.4 ± 0.8	7.1 ± 0.7	0.05
	V−O2	1.4 ± 0.5	2.02 ± 0.04	6.4 ± 0.8	6.4 ± 2.3	

Supplementary Table 5. XPS fitting parameters of binding energies for the as-prepared $Ni_3Fe_{1-x}V_x$ catalysts as well as Fe_2O_3, V_2O_3 and VO_2

Catalyst	Binding energy (eV)								
	Ni^{2+}		Fe^{3+}		V^{3+}		V^{4+}		V^{5+}
	$2p_{1/2}$	$2p_{3/2}$	$2p_{1/2}$	$2p_{3/2}$	$2p_{1/2}$	$2p_{3/2}$	$2p_{1/2}$	$2p_{3/2}$	$2p_{3/2}$
$Ni(OH)_2$	871.1	854.1	–	–	–	–	–	–	–
Ni_3Fe	871.9	854.3	723.4	710.9	–	–	–	–	–
Ni_3V	872.1	854.4	–	–	522.0	514.4	523.1	515.3	516.3
$Ni_3Fe_{0.5}V_{0.5}$	872.3	854.4	724.8	711.5	521.8	514.4	522.8	515.1	516.2
Fe_2O_3 (ref. 1)	–	–	724.6	711.0	–	–	–	–	–
V_2O_3 (ref. 2)	–	–	–	–	523.3	515.7	–	–	–
VO_2 (ref. 2)	–	–	–	–	–	–	523.5	516.2	–

Supplementary Table 6. Binding energies of OH*, O* and OOH* intermediates (E_{OH*}, E_{O*}, E_{OOH*}), theoretical OER overpotential (η_{OER}), rate-limiting step (RLS), and Mulliken charge for various metal sites of the V and Fe doped Ni (oxy)hydroxide (101) surface covered by O, OH or H_2O species[a]

Site	System	E_{OH*} (eV)	E_{O*} (eV)	E_{OOH*} (eV)	η_{OER} (V)	RLS	Charge (e)
V	a-(V,Fe,Ni)_O	0.66	2.48	3.57	0.25	OOH*	1.58
	a-(V,Fe,Ni)_H_2O	0.79	2.66	3.77	0.26	OOH*	1.58
	b-(V,Fe,Ni)_O	0.47	2.70	3.84	0.63	O*	1.65
	c-(V,Fe,Ni)_O	0.66	2.27	3.70	0.59	OOH*	1.67
	(V,Ni)_O	0.73	2.61	3.94	0.49	OOH*	1.64
	(V,Ni)_OH	0.55	2.31	3.53	0.38	OOH*	1.64
	(V,Ni)_H_2O	0.57	2.23	3.48	0.40	OOH*	1.64
Fe	a-(V,Fe,Ni)_O	1.15	3.47	4.05	0.72	O*	0.90
	b-(V,Fe,Ni)_O	1.21	3.57	4.09	0.76	O*	1.08
	(Fe,Ni)_O	1.60	3.84	4.72	0.76	OH*	1.07
	(Fe,Ni)_OH	1.39	3.99	4.32	0.79	O*	1.07
Ni	a-(V,Fe,Ni)_O	1.65	4.20	4.00	0.96	O*	0.76
	(V,Ni)_O	1.73	4.18	4.41	0.89	OH*	0.78
	(Fe,Ni)_O	1.70	4.38	4.34	1.08	O*	0.81
	(Ni)_O	1.58	4.02	4.11	0.84	O*	0.86
	(Ni)_OH	1.82	4.37	4.69	0.98	OH*	0.86
Ru	RuO_2(110)	0.33	2.31	3.65	0.40	OOH*	–

[a] The values of RuO_2 (110) surface are also listed for comparison. The atomic structures of all the models are shown in Fig. 7a and Supplementary Fig. 26. Selected structures of adsorbed O intermediates are shown in Supplementary Fig. 28.

Supplementary Table 7. Comparison of OER performance of $Ni_3Fe_{0.5}V_{0.5}$ NSs with state-of-the-art electocatalysts loaded on a high specific area substrate in 1 M KOH with *iR*-correction

Catalyst	Substrate	J (mA cm^{-2})	η (mV)	Tafel slope (mV dec^{-1})	Bulk electrolysis	Ref.
$Ni_3Fe_{0.5}V_{0.5}$	CFP	10<br>100<br>500	200<br>264<br>291	39	60 h at 100 mA cm^{-2}	This work
IrO_2	CFP	10	264	47	–	3
VOOH	NF[c]	10	270	68	24 h at 20 mA cm^{-2}	4
h-NiS_x	NF	100<br>500	217<br>316	96	10 h at 10 mA cm^{-2}	5
Ni_2P-Ni	NF	100<br>500	268<br>350	–	20 h at 10 mA cm^{-2}	6
CoP/CoO_x	CC	10	281	–	17 h at 10 mA cm^{-2}	3
FeP	CFP	10	350	64	48 h at 15 mA cm^{-2}	7
NiFe NSs	NF	80	270	28	10 h at 100 mA cm^{-2}	8
NiFe[a]	NF	10	240	–	–	9
Fe:$Ni(OH)_2$	NF	500	300	48.5	50 h at η 420 mV[i]	10
$NiFeO_x$	NF	10<br>100	250<br>260	28	self-repair in 1 M KOH/2 M Na_2CO_3 at 0.75 V	11
FeNi-rGO	NF	10	206	39	8 h at 10 mA cm^{-2}	12
NiFe/CNT	CFP[d]	10	235	31	10 h at 10 mA cm^{-2},	13
$NiFeO_x$/CNF NPs	CFP	10<br>100	230<br>280	31.5	100 h at 10 mA cm^{-2}	14
Cu@NiFe	CF[e]	100<br>500	281<br>311	27.8	48 h at 100 mA cm^{-2}	15
NiFe	NF	10	224	53	10 h at 10 mA cm^{-2}	16
NiFe NPs	NF	30	280	50	10 h at 200 mA cm^{-2}	17
Ni-Co NWs	CC[f]	10<br>25	302<br>320	43.6	10 h at 20 mA cm^{-2}	18
NiCo-MOF NSs	CF	10	189	–	200 h at η 250 mV	19
NiCoFe	CC	10	239	32	12 h at 10 mA cm^{-2}	20
$CoFeNiO_x$	NF	10<br>100	240<br>270	32	–	21
$Co_{0.37}Ni_{0.26}Fe_{0.37}O$	CC	10<br>100	232<br>280	37.6	100 h at 20 mA cm^{-2}	22
$Ni_{60}Fe_{30}Mn_{10}$	NF	500	360	62	48 h at 100 mA cm^{-2}	23
Gelled FeCoW[b]	Au-NF[g]	10	191	–	550 h at 30 mA cm^{-2}	24
$Ni_{0.75}Fe_{0.125}V_{0.125}$	NF	10	231	39.4	15 h at 30 mA cm^{-2}	25
$W_{0.5}Co_{0.4}Fe_{0.1}$	NF	10<br>100	250<br>310	32	500 h at 20 mA cm^{-2}	26
$(Ni_{0.75}Fe_{0.25})Se_2$	CC	50<br>100	260<br>277	30.2	28 h at 35 mA cm^{-2}	27
Fe doped NiSe	FNF[h]	100	264	65	20 h at η 270 mV	28
$Ni_xFe_{1-x}Se_2$-DO NSs	NF	100	225	28	24 h at 10 mA cm^{-2}	29
$CoNi_2Se_4$	CFP	10<br>100	160<br>230	72	10 h at η 150 mV	30
$(Ni,Co)Se_{0.85}$-NiCo	CC	10<br>100	216<br>300	77	24 h at 10 mA cm^{-2}	31

[a] In 1 M NaOH. [b] Polarization curves obtained at without *iR*-correction. [c] NF = Ni foam. [d] CFP = carbon fiber paper. [e] CF = Cu foam. [f] CC = carbon cloth. [g] Au-NF = Au-coated Ni foam. [h] FNF = FeNi foam. [i] in 5 M KOH.

Supplementary Table 8. Summary the fitting parameters of *in-situ* Ni, Fe, and V *K*-edge EXFAS curves for $Ni_3Fe_{0.5}V_{0.5}$ NS catalysts

Potential (V versus RHE)	Shell	Coordination number (CN)	Bond distance (R (Å))	ΔE_0 (eV)	σ^2 (10^{-3} Å^2)	R-factor
1.15	Ni−O	6.3 ± 0.4	2.04 ± 0.02	−4.6 ± 1.7	8.0 ± 0.6	0.005
	Ni−M	5.5 ± 0.7	3.12 ± 0.03	−0.3 ± 3.1	8.3 ± 0.8	
	Fe−O	6.2 ± 0.3	2.00 ± 0.01	−2.3 ± 0.6	6.2 ± 0.7	0.002
	Fe−M	5.8 ± 0.9	3.10 ± 0.01	0.8 ± 1.4	8.6 ± 1.2	
	V−O1	3.6 ± 0.5	1.70 ± 0.01	5.0 ± 1.3	4.8 ± 0.7	0.09
	V−O2	2.3 ± 0.8	2.01 ± 0.02	5.0 ± 1.3	4.8 ± 0.7	
1.75	Ni−O	4.7 ± 0.7	1.90 ± 0.04	−6.3 ± 6.7	4.9 ± 0.7	0.008
	Ni−M	3.1 ± 0.9	2.82 ± 0.09	−3.8 ± 11.0	7.4 ± 1.2	
	Fe−O	5.5 ± 0.3	1.97 ± 0.01	1.9 ± 1.8	8.6 ± 1.2	0.08
	Fe−M	5.5 ± 0.6	2.83 ± 0.02	−11.3 ± 3.2	10.8 ± 1.3	
	V−O1	3.9 ± 0.4	1.65 ± 0.01	−6.8 ± 0.9	3.6 ± 0.8	0.46
	V−O2	2.2 ± 0.9	2.01 ± 0.03	−6.8 ± 0.9	5.1 ± 3.1	

Supplementary Table 9. Summary the fitting parameters of the electrochemical impedance spectra of as-prepared $Ni_3Fe_{1-x}V_x$ (oxy)hydroxide catalysts measured at 300 mV overpotential

Catalysts	R_s (Ω)	$R_{ct(int)}$ (Ω)	CPE_1	$R_{ct(s\text{-}l)}$ (Ω)	CPE_2
$Ni(OH)_2$	5.09	1.59	0.37	15.59	0.91
Ni_3Fe	5.05	1.46	0.38	8.55	0.90
Ni_3V	4.92	1.40	0.40	5.78	0.84
$Ni_3Fe_{0.5}V_{0.5}$	5.06	0.65	0.41	3.55	0.75

Supplementary Notes

Supplementary Note 1: More discussion on the calculated $L_{2,3}$-edge XAS spectra of high/low-spin models of Fe^{3+}

We have carried out the calculation of $L_{2,3}$-edge XAS spectra for Fe^{3+} in the FeO_6 octahedral cluster. Parameter values typical for a Fe^{3+} system (U_{dd} = 5.0, Δ = 3.0, and Slater integrals reduced to 75% of Hartree-Fock values) were adopted[32]. Calculations with the parameters ($V_{pd\sigma}$ = −1.35 eV, $V_{pd\pi}$ = 0.52 eV and 10Dq = 1.0 eV) gave rise to the theoretical spectra consistent with the experimental spectra, as shown in Supplementary Fig.17. The ground state of Fe^{3+} stayed a stable high-spin state, which is 1.32 eV lower than the first excited state. The spin state of Fe could be changed to low-spin with increasing the crystal field and the covalent bond. Assuming that $V_{pd\sigma}$ and $V_{pd\pi}$ are fixed, the spin transition between high-spin and low-spin occurred when 10Dq was about 1.9 eV. When 10Dq was increased to 3.0 eV, Fe^{3+} was in a stable low-spin state, which exhibits a quite different line shape with that in high-spin state, as shown in Supplementary Fig.17.

Supplementary Note 2: Estimation of roughness factor (RF) values

The RF of each catalyst was evaluated by electrochemical double-layer capacitances (C_{dl}), which was obtained from the corresponding CV curves measured at varying scan rates (10–100 mV s^{-1}) in the non-faradaic potential region (0.15−0.20 V versus Hg/HgO) in 1 M KOH. From the CVs (Supplementary Fig. 22a–g), the plots of Δj versus scan rate were obtained (Supplementary Fig. 22h), in which the linear slopes are equivalent to twice of the values of C_{dl} at the solid/liquid interface. On the basis of the equation of RF = $C_{dl}/(C_s{\cdot}A_g)$, where C_s is the specific capacitance of CFP (Here C_s = 0.41 mF cm^{-2}) and the A_g is the geometric area of the electrode, the estimated RFs are in an increasing order of $Ni(OH)_2$/CFP (8.2) < Ni_3Fe/CFP (9.4) < Ni_3V/CFP (10.9) < $Ni_3Fe_{0.67}V_{0.33}$/CFP (11.6) < $Ni_3Fe_{0.33}V_{0.67}$/CFP (13.0) < $Ni_3Fe_{0.5}V_{0.5}$/CFP (14.5) (refs 33,34).

Supplementary Note 3: Estimation of the charge transfer resistances

The Nyquist plots (Fig. 5d) are fitted to a simplified Randles equivalent circuit model to obtain the series resistance (R_s), constant-phase element (CPE) and charge-transfer resistance (R_{ct}). The very small semicircles in the high frequency zone are attributed to the internal charge-transfer resistances ($R_{ct(int)}$) of the electrode, and the second semicircles represent the charge-transfer resistance ($R_{ct(s\text{-}l)}$) at the solid/liquid interface between electrode and electrolyte. Both $R_{ct(int)}$ and $R_{ct(s\text{-}l)}$ values

apparently decreased as Fe and V were co-doped into the Ni hydroxide lattice. The total R_{ct} values ($R_{ct} = R_{ct(int)} + R_{ct(s\text{-}l)}$) at 300 mV overpotential are 4.2, 7.2, 10.0, and 17.2 Ω for the CFP-supported $Ni_3Fe_{0.5}V_{0.5}$, Ni_3V, Ni_3Fe, and pure Ni (oxy)hydroxide catalysts, respectively (Supplementary Table 9). The simplified Randles equivalent circuit model can be expressed as follows:

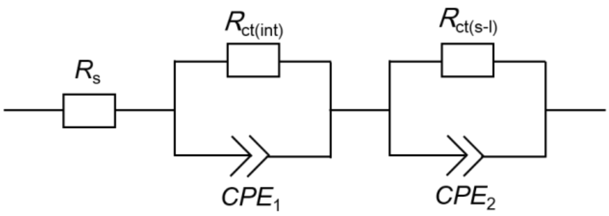

Besides, the corresponding fitting ESI parameters of as-prepared $Ni_3Fe_{1-x}V_x$ (oxy)hydroxide catalysts are summarized in Supplementary Table 9.

Supplementary Note 4: More discussion on Supplementary Fig. 29

At equilibrium potential (U = 0.402 V), the V and Fe co-doped as well as V-doped Ni (oxy)hydroxides show uphill at the second and third steps of OER, and formation of OOH* species (RLS) gives a larger potential step of 0.25 and 0.38 eV, resulting in overpotentials of 0.25 and 0.38 V, respectively. Accordingly, by applying an electrode potential of 0.402 + 0.25 = 0.65 V and 0.402 + 0.38 = 0.78 V for the co-doped and V-doped Ni (oxy)hydroxides, all the elemental steps of OER become downhill and thus can occur spontaneously from the thermodynamic point of view. On the other hand, at U = 0.402 V, the Fe-doped and pristine Ni (oxy)hydroxides show the largest potential step of 0.76 and 0.84 eV during the formation of OH* and O* species (RLS), respectively. Consequently, electrode potentials of 0.402 + 0.76 = 1.16 V and 0.402 + 0.84 = 1.24 V are required for the Fe-doped and pristine Ni (oxy)hydroxides, respectively, to render all the OER steps downhill.

Supplementary Methods

Preparation of $Ni_3Fe_{0.5}V_{0.5}$ (oxy)hydroxide samples for TEM measurements. The $Ni_3Fe_{0.5}V_{0.5}$ (oxy)hydroxide was peeled off from the CFP by continuous ultrasonication in ethanol, and the obtained suspension was dropped onto the carbon-coated copper TEM grids using a micropipettor.

Fabrication of $Ni_3Fe_{0.5}V_{0.5}$ (oxy)hydroxide film electrodes used for the *in-situ* measurements of EC-Raman and XAS. The $Ni_3Fe_{0.5}V_{0.5}$ (oxy)hydroxide powder samples were prepared by the same procedure used for the preparation of $Ni_3Fe_{0.5}V_{0.5}$/CFP, but in the absence of CFP; the product was obtained by centrifugation (10000 rmp, 5 min, HITACHI High-speed Micro Centrifuge, CF 15RN), washed with deionized water and ethanol at least 5 times, and finally dried in a vacuum at 60 ºC overnight for future use. For fabrication of the electrodes used for *in-situ* EC-Raman measurement, the catalyst ink was prepared by dispersing $Ni_3Fe_{0.5}V_{0.5}$ (oxy)hydroxide powder (5 mg) in the solution (1 mL) of deionized water (250 mL) and isopropanol (750 mL) with sonication for 30 min, followed by addition of 5% Nafion (35 μL) to the mixed solution with sonication for another 40 min. Afterwards, the ink (10 μL) was dropped onto a glassy carbon electrode (GCE, 5 mm diameter with a catalyst loading of ~ 0.2 mg cm^{-2}), which was used for the measurement of *in-situ* EC-Raman. The $Ni_3Fe_{0.5}V_{0.5}$/CFP electrode employed for the *in-situ* XAS measurement was fabricated by the same procedure as afore-described, but with one side of CFP covered by a layer of Kapton film (Dongguan Letter Packaging Materials Co., Ltd.) during the hydrothermal process.

EXAFS data analysis. Phase shifts and backscattering amplitudes used for EXAFS data analysis were obtained from the experimental spectra of the reference compounds. After the energy was calibrated, EXAFS data analysis was carried out by using the software package of Ifeffit. The EXAFS function, $\chi(E)$, was obtained by subtracting the post-edge background from the overall absorption and then normalized with respect to the edge jump step. The normalized $\chi(E)$ was transformed from energy space to **κ**-space, where **κ** is the photoelectron wave vector. The $\chi(\boldsymbol{\kappa})$ data were multiplied by $\boldsymbol{\kappa}^2$ to compensate for the damping of EXAFS oscillations in the high **κ**-region. Fourier transformation (FT) from **κ**-space to *R*-space, and the data fit was performed in *R* space. The S_0^2 values of 0.74, 0.74, and 0.95 were obtained from fitting to V, Fe, and Ni standard samples, respectively.

The acquired data before Fourier transform were processed by using the ATHENA module

implemented in the IFEFFIT software packages. For *ex-situ* EXAFS FT, the **k**-rangs of Fourier transform were 3.0–12.4 Å^{-1} for Ni and Fe of Ni_3Fe and $Ni_3Fe_{0.5}V_{0.5}$, 2.8–12.0 Å^{-1} for V of Ni_3V, and 3.0–12.5 Å^{-1} for V of $Ni_3Fe_{0.5}V_{0.5}$. The quantitative curve-fittings were performed in the *R*-space range of 1.0–3.3 Å for Ni, 1.0–3.5 Å for Fe, 0.9–2.0 Å for V of Ni_3V, and 0.7–2.0 Å for V of $Ni_3Fe_{0.5}V_{0.5}$, respectively. The Hanning window ($\Delta\mathbf{k}$ = 1.0 Å^{-1}) was used as the window function. Furthermore, for *in-situ* EXAFS FT, the **k**-rangs of Fourier transform were 2.8–9.3 Å^{-1} for Ni, 3.0–10.5 Å^{-1} for Fe, 2.8–10.0 Å^{-1} for V of $Ni_3Fe_{0.5}V_{0.5}$, and the quantitative curve-fittings were performed in the *R*-space range of 1.0–3.3 Å for Ni, 1.0–3.0 Å for Fe, 0.7–2.2 Å for V at 1.15 V, and 0.5–2.1 Å for V of $Ni_3Fe_{0.5}V_{0.5}$ at 1.75 V.

The FT curves shown in Figs 3 and 6, as well as in Supplementary Figs 8, 11, 12, 13, and 14, are not phase-shift corrected, and hence the peak position is shorter than the real interatomic distance by an amount of approximately 0.3−0.5 Å depending on the type of neighboring atoms. The accurate bond lengths were obtained from curve-fitting.

The uncertainties of the best-fit parameters were estimated by the Artemis code of the Ifeffit software. Based on the appropriate estimation of the uncertainty of the EXAFS measurement, the uncertainty in the table of EXAFS results estimated by Ifeffit is the error bar of the fitting parameter.

The phase-shift functions of Fe–O, Fe–Ni, V–O, and V–Ni pairs were obtained by FEFF calculations based on the structural models of a Fe (or V) atom substituting for a Ni site in $Ni(OH)_2$ lattice. The data of $Ni_3Fe_{0.5}V_{0.5}$ were fitted by using two V–O paths which yield better matches between the experimental data and fitting curves. More reliable structural parameters are obtained. It helps us to confirm the significantly larger local distortion of the octahedron around V than around Fe and Ni, with the obvious V–O bond length splitting.

Calculation of the $L_{2,3}$-edge XAS spectra of high/low-spin models of Fe^{3+}. The Fe $L_{2,3}$-edge XAS spectra were simulated with a configuration interaction cluster model which includes the full atomic multiplet theory and the hybridization with the O 2*p* ligands[35]. Ligand field multiplet calculations were performed using the multiplet model implemented by Thole[36], the atomic theory developed by Cowan[37] and the crystal field interactions described by Butler[38]. Both electronic Coulomb interactions and spin-orbit coupling for each sub-shell are considered. Hartree-Fock estimates the radial part of matrix elements of the Coulomb interaction in terms of Slater integrals F^k and G^k and

the spin-orbit coupling parameters ζ(3*d*) and ζ(2*p*) for the core 2*p* and valence 3*d* shells. As a donor ligand system, hybridization between Fe-3*d* orbit and O-2*p* orbit is treated using a charge transfer model, in which $3d^6\underline{L}$ and $3d^7\underline{L}$ configurations above the $3d^5$ configurations are involved. The energy difference of these configurations is defined by the charge transfer energy Δ and the 3*d*-3*d* Coulomb interaction U_{dd}. The ground state can be written as $\varphi = \alpha|3d^5> + \beta|3d^6\underline{L}> \gamma|3d^7\underline{L}>$, where the coefficients α, β and γ are determined by the Hamiltonian:

$$H = \begin{vmatrix} 0 & T & 0 \\ T & \Delta & T \\ 0 & T & 2\Delta + U_{dd} \end{vmatrix}$$

The spectrum is calculated from the sum of all possible transition for an electron excited from the 2*p* level into a 3*d* level.

EIS and RF measurements and analyses. The Nyquist plots were measured with an electrochemical workstation (Zahner zennium/IM6, Germany) at a bias of 300 mV with the sweeping of frequency from 100 kHz to 0.1 Hz and an AC amplitude of 5 mV. The capacitive currents ($\Delta j = j_a - j_c$), measured at 0.175 V versus Hg/HgO, were plotted as a function of scan rate (v), and the C_{dl} values were determined by the linear slopes in the Δj–v plots. The C_{dl} can be further converted into RF using the equation of RF = $C_{dl}/(C_s \cdot A_g)$, where C_s is the specific capacitance of CFP and A_g is the geometric area of the electrode[34]. Here we employed the specific capacitance for the pristine CFP in 1 M KOH as C_s, which is 0.41 mF cm^{-2} in our measurement.

Turnover frequency (TOF) calculation. The TOF values were calculated as the number of oxygen molecules evolved per active site per second based on the following equation:

$$\text{TOF} = \frac{J \times A \times FE}{4F \times m}$$

Where J is the current density (A cm^{-2}) at a given overpotential, A is the effective surface geometric area of the working electrode (0.2 cm^2), FE is the Faradaic efficiency, F is the Faraday constant, and m is the number of moles of the active metal on the electrode. In TOF calculations, we assumed that all Ni, Fe, and V atoms in $Ni_3Fe_{1-x}V_x$ were accessible for catalyzing the OER.

Models for DFT calculation and binding energies of oxygen intermediates. We consider the (101) surface of β-NiOOH for OER catalysis because (i) the PXRD and HRTEM experiments on

the as-prepared $Ni_3Fe_{0.5}V_{0.5}$ show that the (101) surface is the dominant exposed surface of $Ni(OH)_2$, (ii) previous theoretical and experimental studies demonstrated that the (oxy)hydroxide phase of LDH is the stable phase under typical OER conditions in alkaline media[10,14,33,39], and (iii) in our case NiOOH has been detected by *in-situ* Raman and XAS spectroscopies for the activated $Ni_3Fe_{0.5}V_{0.5}$ catalyst. A slab model consisting of four metal layers was used with a vacuum regime of 16 Å in the vertical direction (Fig. 7a and Supplementary Fig. 26). The lateral dimension includes 2 × 2 unit cells comprising eight Ni atoms per layer; two of them were substituted by V and Fe atoms, respectively. For comparison, we also considered β-NiOOH doped by V or by Fe atom only. The supercell consists of 2 × 1 unit cells for the lateral direction. One of the four Ni atoms on each layer was replaced by V (Fe) atom, corresponding to Ni:V (Fe) ratio of 3:1. The Brillouin zones of the two types of supercells were sampled by 3 × 3 × 1 and 3 × 6 × 1 uniform **k**-point mesh. The model structures were optimized by the ionic and electronic degrees of freedom using thresholds for the total energy of 10^{-4} eV and the force of 0.02 eV $Å^{-1}$. The lattice parameters for all the models were fixed at the experimental value (a = 3.08 Å). During the structural relaxation, the bottom layer H, O and metal atoms were fixed to mimic a semi-infinite solid.

For the V and Fe co-doped β-NiOOH, we considered three models with V and Fe atoms in different relative positions, as shown in Supplementary Fig. 26a–c. Model a- and c-(NiFeV)-OOH, have some V and Fe atoms aggregated, and they are lower in energies by 0.36 and 0.44 eV, respectively, than model b-(NiFeV)-OOH with uniform doping. For all the models, the surface metal atoms except the reaction site are covered by O, OH, or H_2O species[40,41]. The binding energies of oxygen intermediates are defined as

$$E_{OH*} = E_{OH*+cat.} - E_{cat.} - (E_{H2O} - 0.5E_{H2}) \quad (1)$$

$$E_{O*} = E_{O*+cat.} - E_{cat.} - (E_{H2O} - E_{H2}) \quad (2)$$

$$E_{OOH*} = E_{OOH*+cat.} - E_{cat.} - (2E_{H2O} - 1.5E_{H2}) \quad (3)$$

where $E_{OH*+cat.}$, $E_{O*+cat.}$, and $E_{OOH*+cat.}$ are the energies of an OH*, O*, and OOH* species adsorbed on the catalyst material, respectively; $E_{cat.}$ is the energy of the catalyst with a clean surface; E_{H2O} and E_{H2} are the energies of a H_2O and H_2 molecule in vacuum, respectively.

Gibbs free energies and theoretical overpotentials. The four-electron pathway in alkaline media for OER was considered as follows[40,42]:

$$* + OH^{-} \rightarrow OH* + e^{-} \quad (4)$$

$$OH^* + OH^- \rightarrow O^* + H_2O\ (l) + e^- \quad (5)$$

$$O^* + OH^- \rightarrow OOH^* + e^- \quad (6)$$

$$OOH^* + OH^- \rightarrow * + O_2\ (g) + H_2O\ (l) + e^- \quad (7)$$

The Gibbs free energy of formation for each reaction step is given by

$$\Delta G = \Delta E_{DFT} + \Delta ZPE - T\Delta S - eU \quad (8)$$

where ΔE_{DFT} is the total energy difference between the final and the initial states; ΔZPE is the zero point vibrational energy; T is temperature and taken as 298 K; ΔS is the entropy; e is the charge transfer; U is the electrode voltage. The ΔZPE and ΔS values of H_2O, H_2, and O_2 molecules were taken from the NIST-JANAF thermodynamics table[43], with $\Delta ZPE - T\Delta S$ equal to –0.11, –0.14, and –0.53 eV, respectively. As for the oxygen intermediates species, the vibrational frequencies were calculated on selected metal sites of the Ni-based (oxy)hydroxide models, and they gave similar results for $\Delta ZPE - T\Delta S$ equal to 0.30, 0.32, and 0.05 eV for OH*, OOH*, and O* species, respectively.

Within the SHE method, the Gibbs free energy difference of OH^- and e^- can be calculated as follows[44]:

$$G(OH^-) - G(e^-) = G(H_2O) - (1/2)G(H_2) + k_BT\ln 10\cdot pH \quad (9)$$

where the last term is the correction to the Gibbs free energy for OH^- anions at pH other than 0 (here pH = 14); k_B is the Boltzmann constant. Therefore, the Gibbs free energy of formation for each OER step can be obtained by calculating the energy (or binding energy) of OH*, O*, and OOH* intermediates adsorbed on the catalyst surface. Eventually, the theoretical OER overpotential can be evaluated by the following equation:

$$\eta_{OER} = \Delta G_{max}/e - 0.402\ V \quad (10)$$

where ΔG_{max} is the maximum value of ΔG among the four OER steps; 0.402 V is the equilibrium voltage at pH = 14 and T = 298 K (ref. 45).

Supplementary References

1. McIntyre, N. S. & Zetaruk, D. G. X-ray photoelectron spectroscopic studies of iron oxides. *Anal. Chem.* **49,** 1521–1529 (1977).
2. Sawatzky, G. A. & Post, D. X-ray photoelectron and Auger spectroscopy study of some vanadium oxides. *Phys. Rev. B* **20**, 1546–1555 (1979).
3. Wang, P., Song, F., Amal, R., Ng, Y. H. & Hu, X. Efficient water splitting catalyzed by cobalt phosphide-based nanoneedle arrays supported on carbon cloth. *ChemSusChem* **9**, 472–477 (2016).
4. Shi, H., Liang, H., Ming, F. & Wang, Z. Efficient overall water-Splitting electrocatalysis using lepidocrocite VOOH hollow nanospheres. *Angew. Chem. Int. Ed.* **56**, 573–577 (2017).
5. You, B. & Sun, Y. Hierarchically porous nickel sulfide multifunctional superstructures. *Adv. Energy Mater*. **6**, 1502333 (2016).
6. You, B., Jiang, N., Sheng, M., Bhushan, M. W. & Sun, Y. Hierarchically porous urchin-like Ni_2P superstructures supported on nickel foam as efficient bifunctional electrocatalysts for overall water splitting. *ACS Catal.* **6,** 714–721 (2016).
7. Xiong, D., Wang, X., Li W. & Liu, L. Facile synthesis of iron phosphide nanorods for efficient and durable electrochemical oxygen evolution. *Chem. Commun*. **52**, 8711–8714 (2016).
8. Lu, X. & Zhao, C. Electrodeposition of hierarchically structured three-dimensional nickel–iron electrodes for efficient oxygen evolution at high current densities. *Nat. Commun.* **6,** 6616 (2015).
9. Zhang, W., Qi, J., Liu, K. & Cao, R. A nickel-based integrated electrode from an autologous growth strategy for highly efficient water oxidation. *Adv. Energy Mater.* **6**, 1502489 (2016).
10. Luo, J. *et al*. Water photolysis at 12.3% efficiency via perovskite photovoltaics and Earth-abundant catalysts. *Science* **345**, 1593–1596 (2014).
11. Wang, J., Ji, L. & Chen, Z. In situ rapid formation of a nickel−iron-based electrocatalyst for water oxidation. *ACS Catal.* **6**, 6987–6992 (2016).
12. Long, X. *et al*. A strongly coupled graphene and FeNi double hydroxide hybrid as an excellent electrocatalyst for the oxygen evolution reaction. *Angew. Chem. Int. Ed.* **53,** 7584–7588 (2014).
13. Gong, M. *et al*. An advanced Ni−Fe layered double hydroxide electrocatalyst for water oxidation. *J. Am. Chem. Soc.* **135**, 8452–8455 (2013).
14. Wang, H. *et al*. Bifunctional non-noble metal oxide nanoparticle electrocatalysts through lithium-induced conversion for overall water splitting. *Nat. Commun.* **6**, 7261 (2015).
15. Yu, L. *et al.* Cu nanowires shelled with NiFe layered double hydroxide nanosheets as bifunctional electrocatalysts for overall water splitting. *Energy Environ. Sci.* **10**, 1820–1827

(2017).

16. Li, Z. *et al.* Fast electrosynthesis of Fe-containing layered double hydroxide arrays toward highly efficient electrocatalytic oxidation reactions. *Chem. Sci.* **6**, 6624–6631 (2015).
17. Lu, Z. *et al.* Three-dimensional NiFe layered double hydroxide film for high-efficiency oxygen evolution reaction. *Chem. Commun*. **50**, 6479–6482 (2014).
18. Bae, S. -H. *et al.* Seamlessly conductive 3D nanoarchitecture of core–shell Ni-Co nanowire network for highly efficient oxygen evolution. *Adv. Energy Mater.* **7**, 1601492 (2017).
19. Zhao, S. *et al.* Ultrathin metal–organic framework nanosheets for electrocatalytic oxygen evolution. *Nat. Energy* **1**, 16184 (2016).
20. Wang, A. -L., Xu, H. & Li, G. -R. NiCoFe layered triple hydroxides with porous structures as high-performance electrocatalysts for overall water splitting. *ACS Energy Lett.* **1,** 445–453 (2016).
21. Morales-Guio, C. G., Liardet, L. & Hu, X. Oxidatively electrodeposited thin-film transition metal (oxy)hydroxides as oxygen evolution catalysts. *J. Am. Chem. Soc.* **138,** 8946–8957 (2016).
22. Chen, W. *et al*. In situ electrochemical oxidation tuning of transition metal disulfides to oxides for enhanced water oxidation. *ACS Cent. Sci.* **1**, 244–251 (2015).
23. Detsi, E. *et al.* Mesoporous $Ni_{60}Fe_{30}Mn_{10}$-alloy based metal/metal oxide composite thick films as highly active and robust oxygen evolution catalysts. *Energy Environ. Sci.* **9,** 540–549 (2016).
24. Zhang, B. *et al.* Homogeneously dispersed multimetal oxygen-evolving catalysts. *Science* **352,** 333–337 (2016).
25. Dinh, K. N. *et al.* Ultrathin porous NiFeV ternary layer hydroxide nanosheets as a highly efficient bifunctional electrocatalyst for overall water splitting. *Small* 1703257 (2017).
26. Pi, Y. *et al.* Trimetallic oxyhydroxide coralloids for efficient oxygen evolution electrocatalysis. *Angew. Chem. Int. Ed.* **56,** 4502–4506 (2017).
27. Wang, Z. *et al*. Porous nickel–iron selenide nanosheets as highly efficient electrocatalysts for oxygen evolution reaction. *ACS Appl. Mater. Interfaces* **8**, 19386–19392 (2016).
28. Tang, C., Asiri, A. M. & Sun, X. Highly-active oxygen evolution electrocatalyzed by a Fe-doped NiSe nanoflake array electrode. *Chem. Commun.* **52**, 4529–4532 (2016).
29. Xu, X., Song, F. & Hu, X. A nickel iron diselenide-derived efficient oxygen-evolution catalyst. *Nat. Commun.* **7,** 12324 (2016).
30. Amin, B. G., Swesi, A. T., Masud, J. & Nath, M. $CoNi_2Se_4$ as an efficient bifunctional electrocatalyst for overall water splitting. *Chem. Commun.* **53,** 5412–5415 (2017).
31. Xia, C., Jiang, Q., Zhao, C., Hedhili, M. N. & Alshareef, H. N. Selenide-based electrocatalysts

and scaffolds for water oxidation applications. *Adv. Mater.* **28**, 77–85 (2016).

32. Kuo, C.-Y. *et al.* $k = 0$ magnetic structure and absence of ferroelectricity in $SmFeO_3$. *Physical Review Letters* **113,** 217203–217208 (2014).
33. Liang, H., Gandi, A. N., Anjum, D. H., Wang, X., Schwingenschlögl, U. & Alshareef, H . N. Plasma-assisted synthesis of NiCoP for efficient overall water splitting. *Nano Lett.* **16**, 7718−7725 (2016).
34. McCrory, C. C. L., Jung, S., Peters, J. C. & Jaramillo, T. F. Benchmarking heterogeneous electrocatalysts for the oxygen evolution reaction. *J. Am. Chem. Soc.* **135,** 16977–16987 (2013).
35. de Groot, F. M. F. X-ray absorption and dichroism of transition metals and their compounds. *J. Electron Spectrosc. Relat. Phenom.* **67,** 529–622 (1994).
36. Thole, B. T. *et al.* 3*d* x-ray-absorption lines and the $3d^9 4f^{n+1}$ multiplets of the lanthanides. *Phys. Rev. B* **32,** 5107–5118 (1985).
37. Cowan, R. D. *The Theory of Atomic Structure and Spectra* (Univ. of California Press, Berkeley, 1981).
38. Butler, P. H. *Point Group Symmetry Applications: Methods and Tables* (Springer Science & Business Media, 2012).
39. Subbaraman, R. *et al.* Trends in activity for the water electrolyser reactions on 3*d* M(Ni,Co,Fe,Mn) hydr(oxy)oxide catalysts. *Nat. Mater.* **11,** 550−557 (2012).
40. Li, Y. & Selloni, A. Mechanism and activity of water oxidation on selected surfaces of pure and Fe-doped NiO_x. *ACS Catal.* **4,** 1148–1153 (2014).
41. García-Mota, M., *et al.* Importance of correlation in determining electrocatalytic oxygen evolution activity on cobalt oxides. *J. Phys. Chem. C* **116,** 21077–21082 (2012).
42. Rossmeisl, J., Logadottir, A. & Nørskov, J. K. Electrolysis of water on (oxidized) metal surfaces. *Chem. Phys.* **319,** 178–184 (2005).
43. Chase, M. W. NIST-JANAF Thermochemical Tables, American Institute of Physics for the National Institute of Standards and Technology, New York, NY, USA (1998).
44. Nørskov, J. K. *et al.* Origin of the overpotential for oxygen reduction at a fuel-cell cathode. *J. Phys. Chem. B* **108,** 17886–17892 (2004).
45. Zhou, S., Liu, N., Wang, Z. & Zhao, J. Nitrogen-doped graphene on transition metal substrates as efficient bifunctional catalysts for oxygen reduction and oxygen evolution reactions. *ACS Appl. Mater. Interfaces* **9,** 22578–22587 (2017).
